# Meta-analysis of the Vmp-like sequences of Lyme disease *Borrelia*: evidence for the evolution of an elaborate antigenic variation system

**DOI:** 10.3389/fmicb.2024.1469411

**Published:** 2024-10-10

**Authors:** Steven J. Norris, Kalvis Brangulis

**Affiliations:** ^1^Department of Pathology and Laboratory Medicine, McGovern Medical School, University of Texas Health Science Center at Houston, Houston, TX, United States; ^2^Department of Microbiology and Molecular Genetics, McGovern Medical School, University of Texas Health Science Center at Houston, Houston, TX, United States; ^3^Department of Human Physiology and Biochemistry, Faculty of Medicine, Rīga Stradiņš University, Riga, Latvia; ^4^Latvian Biomedical Research and Study Centre, Riga, Latvia

**Keywords:** Lyme disease, *Borrelia*, *vlsE*, antigenic variation, immune evasion, genetics

## Abstract

VMP-like sequence (*vls*) antigenic variation systems are present in every Lyme disease *Borrelia* strain with complete genome sequences. The linear plasmid-encoded *vls* system consists of a single expression site (*vlsE*) and contiguous array(s) of silent cassettes that have ~90% identity with the central cassette region of the cognate *vlsE* gene; antigenic variation occurs through random, segmental, and unidirectional recombination of *vls* silent cassette sequences into the *vlsE* expression site. Automated annotation programs do not accurately recognize *vls* silent cassette sequences, so these regions are not correctly annotated in most genomic sequences. In this study, the *vls* sequences were re-analyzed in the genomic sequences of 31 available Lyme disease *Borrelia* and one relapsing fever *Borrelia* organisms, and this information was utilized to systematically compare the *vls* systems in different species and strains. In general, the results confirm the conservation of the overall architecture of the *vls* system, such as the head-to-head arrangement of *vlsE* and a contiguous series of *vlsS* silent cassette sequences and presence of inverted repeat sequences between the two regions. However, the data also provide evidence for the divergence of the *vls* silent cassette arrays through point mutations, short indels, duplication events, and rearrangements. The probable occurrence of convergent evolution toward a *vls* system-like locus is exemplified by *Borrelia turcica*, a variable large protein (Vlp) expressing organism that is a member of the relapsing fever *Borrelia* group.

## 1 Introduction

The Lyme disease *Borrelia,* also known as *Borrelia burgdorferi sensu lato* (s.l.) (Margos et al., 2018; Margos et al., 2020a) or the genus *Borreliella* (Adeolu and Gupta, 2014; Gupta, 2019), are a group of closely related spirochetes that cause Lyme disease (LD) in humans and other mammals (Radolf and Samuels, 2021; Smith, 2022). The life cycle of these organisms, as exemplified by the species *B. burgdorferi sensu stricto* (s.s.), consists of sequential passage between ticks of the genus *Ixodes* and mammals, without the existence of any other natural reservoirs. In humans, infection occurs following the bite of an infected tick and commonly results in an expanding, localized skin lesion called erythema migrans. The bacteria can rapidly disseminate to other tissues, often causing a range of neurologic, arthritic, and cardiovascular manifestations that may persist long-term. LD *Borrelia* spirochetes can survive in ticks and mammals for months to years, indicating the evolution of mechanisms to evade the immune responses in both the arthropod and mammalian hosts. The pattern of long-term persistence, dissemination, and pathogenesis through the induction of host inflammatory responses are hallmarks of Lyme borreliosis (Coburn et al., 2021).

**Figure 1:**
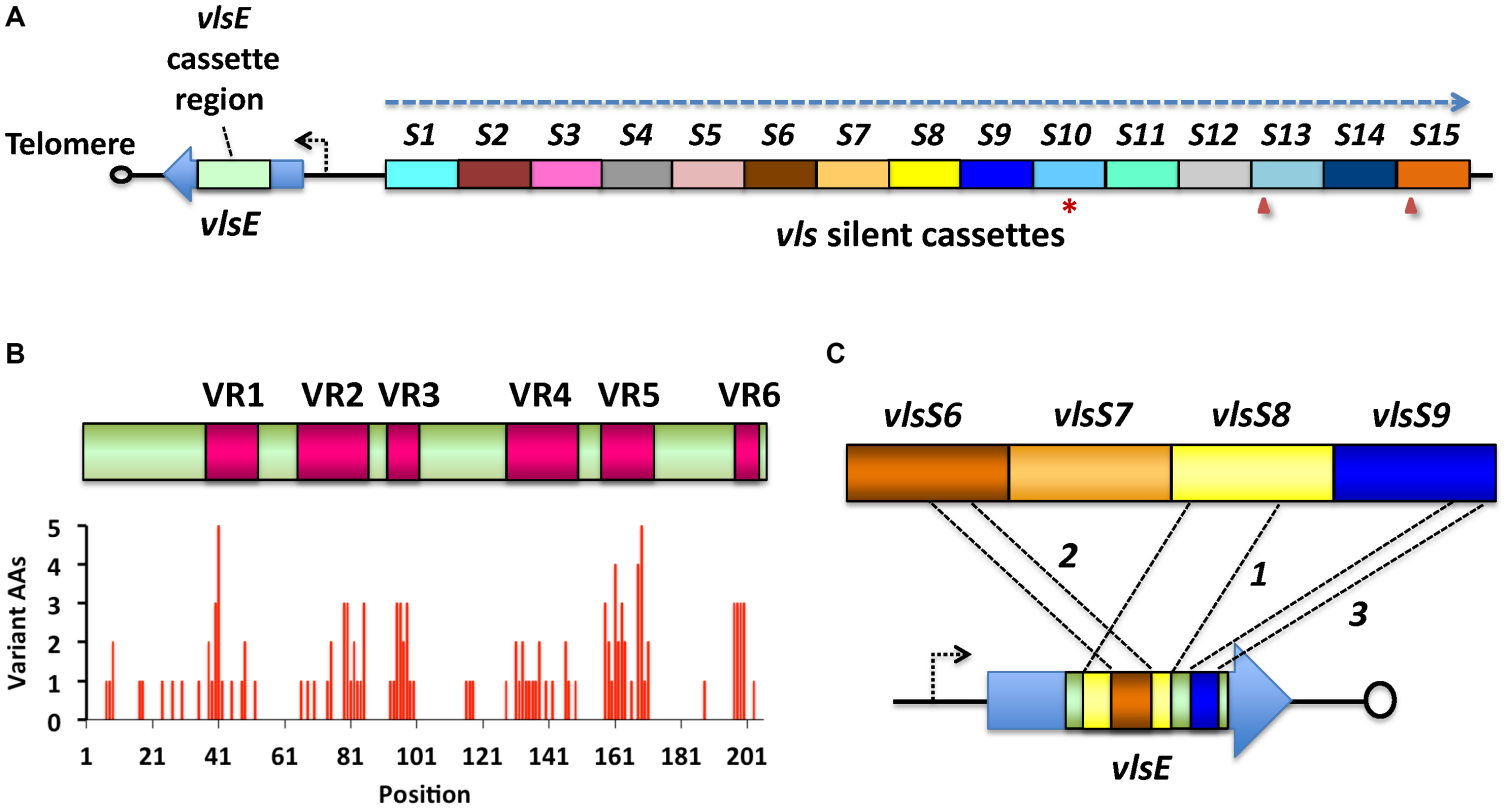
The *vls* system of Lyme disease *Borrelia*, as exemplified by *B. burgdorferi* B31. **(A)** The *vls* locus consists of the *vlsE* gene (expressing a 34-kDa surface lipoprotein), a short intervening sequence, and a contiguous array of silent cassettes (*vlsS1* through *vlsS15*, labeled *S1-S15* here) that are ~90% identical to the central cassette region of *vlsE*. The locus is on a linear plasmid and is typically close to one of the telomeres; *vlsE* and the silent cassette array are in a head-to-head arrangement, with their reading frames running in opposite directions (dashed arrow). The silent cassette array may contain frameshifts (arrowheads) or stop codons (asterisks) either within or between the cassettes. **(B)** Alignment of the *vlsE* cassette region and the silent cassettes reveals the presence of six variable regions (VRs) separated by six relatively invariant regions (IRs). Sequence differences are concentrated in the VRs. **(C)** During mammalian infection, sequential gene conversion events result in the replacement of portions of the *vlsE* cassette region, resulting in changes in the VlsE amino acid sequence and hence antigenic variation. In this hypothetical example, a large segment of *vlsS8* first replaces part of the *vlsE* cassette region, followed by gene conversion events from *vlsS6* and *vlsS9*. The recombinations can range in size from a few base pairs to nearly the full length of the cassette region, and may occur in any location, potentially resulting in >10^40^ possible amino acid combinations (Zhang et al., 1997; Zhang and Norris, 1998a; Coutte et al., 2009; Verhey et al., 2018a). Adapted from Norris (2014).

A wide variety of bacterial and protozoal pathogens evade the immune response by a mechanism called antigenic variation (Seifert and So, 1988; van der Woude and Bäumler, 2004; Palmer and Brayton, 2007; Vink et al., 2012; Palmer et al., 2016). In this process, the pathogen employs specialized genetic or epigenetic mechanisms to rapidly change their surface structure, thus staying “one step ahead” of antibody and T-cell responses. The antigenic variation system found in Lyme disease *Borrelia* is called the Variable Major Protein-like-system (*vls*), based on the similarity of its expressed lipoprotein, VlsE, to the Variable large protein (Vlp) of relapsing fever *Borrelia* (Norris, 2014; Bankhead, 2016; Chaconas et al., 2020). The *vls* system was first described in *B. burgdorferi* B31, in which the 28 kb linear plasmid lp28-1 encodes a single *vlsE* expression site located near one telomere and a contiguous array of 15 silent cassettes (here called *vlsS1* through *vlsS15*) located just upstream of *vlsE* (Zhang et al., 1997); this system is depicted in Figure 1. The silent cassettes and the corresponding cassette region of *vlsE* have roughly 90% sequence identity with each other, with most of the sequence differences concentrated in 6 regions called variable regions (VRs). Sequence variation in *vlsE* and its encoded protein occurs through unidirectional gene conversion events between the silent cassettes and the cassette region of *vlsE*. These recombination events are random in length and location within the *vlsE* cassette region and occur frequently and continuously during mammalian infection (Coutte et al., 2009; Verhey et al., 2018a, 2018b). The expression of major surface lipoproteins by *B. burgdorferi* progresses from Outer surface proteins (Osp) OspA and OspB in the unfed tick to OspC during the tick feeding and transmission phase, followed by VlsE after the first few days of mammalian infection (Liang et al., 2004; Tilly et al., 2013; Coburn et al., 2021; Stevenson, 2023). The regulatory protein YebC has been shown to play a major role in controlling *vlsE* gene transcription (Zhang et al., 2020). VlsE expression and sequence variation are required for survival of *B. burgdorferi* in immunocompetent mouse models, whereas VlsE-deficient spirochetes are able to infect *Rag1^−/−^* immunodeficient mice for long periods (Purser and Norris, 2000; Lawrenz et al., 2004; Bankhead and Chaconas, 2007; Rogovskyy et al., 2017). Thus, the *vls* antigenic variation system plays a key role in the battle between Lyme disease *Borrelia* and the adaptive immune response of mammals, permitting its persistence in reservoir animals, which in turn leads to long-term manifestations such as neuroborreliosis, Lyme arthritis, and acroderma chronicum atrophicans (ACA) in humans. VlsE properties that have been hypothesized to be important in host-pathogen interactions include blockage of antibody binding to invariant surface proteins (Lone and Bankhead, 2020), dermatan sulfate binding (Tan et al., 2022), and homodimer formation (Verhey et al., 2019). Despite its role in immune evasion, VlsE elicits a robust antibody response in the mammalian host, particularly against a relatively invariant region called IR6 or C6 (Lawrenz et al., 1999; Liang et al., 1999). The specific and sensitive immunoreactivity of VlsE or the C6 peptide have led to their utilization in many immunodiagnostic tests for Lyme disease (Bacon et al., 2003; Marangoni et al., 2008; Branda and Steere, 2021).

Despite their importance in the pathogenesis of Lyme disease *Borrelia*, *vls* systems are only well characterized in a small number of species and strains, including *B. burgdorferi* B31 (Zhang et al., 1997; Zhang and Norris, 1998a, 1998b), *B. burgdorferi* JD1 (Verhey et al., 2019), *B. garinii* Ip90, and *B. afzelii* ACA-1 (Wang et al., 2003). A major hindrance to the characterization of the *vls* system in additional Lyme disease *Borrelia* strains is the inability of automated annotation systems to effectively identify and describe *vls* silent cassettes and *vlsE* expression sites. In this study, we analyzed a set of 31 genomic sequences for the presence of *vls* systems and described the locations and arrangements of *vlsE* and *vlsS* elements, as well as associated genetic features such as inverted repeats. In this process, we identified several *vls* loci that provide insights into the evolutionary process that led to the development and diversification of this intricate antigenic variation mechanism. Examples consistent with the early developmental stages of similar systems in Lyme disease and relapsing fever *Borrelia* are also described.

## 2 Materials and methods

### 2.1 Identification and characterization of *vls* sequences

All sequences used in this study were obtained from the National Center for Biomedical Information (NCBI) website.^1^ The strains, replicons, and accession numbers for the *vls* silent cassette regions are listed in Table 1, and those for the *vlsE* sequences are listed in Supplementary Table S1. Another gene family related to *vlsE* called *vls* homolog (*vlsH*) is also found in some Lyme disease *Borrelia* (Norris, 2014); members of this gene family are listed in Supplementary Table S2. The identity of *vlsE* sequences were verified by their high sequence identity to known *vlsE* sequences (e.g., *B. burgdorferi* B31 U76405), including the presence of *vlsE*-specific regions both 5′ and 3′ of the central cassette region. *vlsS* silent cassettes are frequently annotated as variable large family protein, *vlsE*, lipoprotein, or hypothetical protein genes, necessitating a global search for these sequences. Typically, tblastn was performed using either an identified VlsE sequence from the strain under investigation, VlsE from a closely related strain, or the B31 VlsE1 protein sequence (AAC45733.1) to initially identify silent cassette regions. Excluding *B. burgdorferi* B31 (for which several thousand *vlsE* variant sequences have been annotated) from the tblastn search aided in this process. The corresponding replicon or contig sequences were downloaded for further analysis. Tblastn or other blast analyses typically provide only partial alignments with *vlsS* cassettes due to the frequent occurrence of indels, frameshifts, and other sequence differences in the six variable regions (VRs). The 5′ and 3′ ends of each *vlsS* cassette therefore must be determined by an iterative process involving mapping of the *vlsS* candidates using programs such as DNASTAR Lasergene SeqBuilder (DNAStar, Inc. Madison, Wisconsin, United States), MAFFT alignments (Katoh et al., 2019), and manual editing and alignment. The identification of the 5′ and 3′ ends is aided by alignment with *vlsE* sequences, which contain unique “non-cassette” sequences at either end. In some strains, frameshifts occurring between neighboring cassettes helped define the cassette structure. *vlsE* and *vlsS* cassette coordinates and maps are available in Supplementary Files 1, 2.

**Table 1:** Summary of *Borrelia vls* system loci described in this study.

Strain	Plasmid	NCBI accession no.	Ref.	*vlsE* identified	Telomere proximal?	No. of intracassette FS/intercassette FS/intracassette SC/intercassette SC^a^	No. of silent cassettes	Total *vlsS* region length (bp)	G + C (%)	GC Skew	AT Skew
*B. burgdorferi*											
B31	lp28-1	NC_001851.2	Zhang et al. (1997)	Yes	Yes	2/0/1/0	15	8,161	49.9	0.554	0.046
64b	lp28-1	NC_012168.1 CP001419.1	Schutzer et al. (2011)	Partial	Unknown	4/0/0/0	22^b^	12,174	49.7	0.555	0.045
94a	lp28-8	NZ_ABGK02000011.1 NZ_ABGK02000012.1 ABGK02000010.1	–	Partial	Unknown	0/0/0/0	7 + 7 = 14^b,c^	8,109	48.6	0.500	−0.077
118a	lp32-3	CP001530.1	Schutzer et al. (2011)	No	Unknown	1/5/0/3	8^b^	4,609	48.2	0.503	0.061
156a	lp28-1	NC_011864.1	Schutzer et al. (2011)	No	Unknown	0/7/0/0	16^b^	9,103	48.6	0.555	0.016
297	lp28-1	NC_018988.1 AY052626.1	Schutzer et al. (2011)	Separate	Unknown	2/8/0/1	12^b^	5,972	47.7	0.561	0.030
29,805	lp36	NC_012498.1	Schutzer et al. (2011)	No	Unknown	0/0/0/0	17	9,693	48.2	0.498	−0.088
B17/2013	pGr-39_lp30	CP077735.1	–	Yes	Yes	0/1/0/0	18	10,988	48.2	0.495	−0.059
Bol26	lp28-3	NC_012497.1	Schutzer et al. (2011)	No	Unknown	0/4/0/0	14	7,610	48.8	0.539	−0.022
JD1	lp28-1	NC_017404.1 MH509399.1	Schutzer et al. (2011)	Yes	Yes	0/7/0/0	14	7,674	48.1	0.549	0.008
MM1	lp28-8	CP031409.1	Loken et al. (1985), Jabbari et al. (2018)	Yes	Yes	0/3/0/0	15	8,957	48.8	0.510	−0.069
cN40	lp36	NC_017414.1	Schutzer et al. (2011)	No	Unknown	0/4/1/2	19	7,243	48.4	0.510	−0.092
PAbe	lp28-1	CP019923.1	–	Yes	Yes	3/0/1/0	15	8,168	49.8	0.553	0.045
WI91-23	lp28-1	CP001456.1	Schutzer et al. (2011)	No	Unknown	2/0/0/0	16	9,234	49.1	0.582	0.092
ZS7	lp28-1	NC_011780.1	Schutzer et al. (2011)	No	Unknown	4/0/0	12^b^	6,343	49.9	0.548	0.051
***B. garinii***											
A87S	– (partial)	AF274070.1	Wang et al. (2001)	No	Unknown	0/0/0/0	4^b^	1983	51.3	0.472	−0.123
Far04	lp28-1	NC_011873.1	Casjens et al. (2011)	Yes	Yes	2/9/0/0	19	9,792	48.6	0.548	−0.007
IP90	lp28	AY100633.1	Wang et al. (2003)	Yes^d^	Yes^e^	0/0/0/0	11^b^	5,109	49.1	0.522	−0.009
***B. afzelii***											
ACA1	lp28	AY100628.1	Wang et al. (2003)	No^d^	No	1/0/0/0	14^b^	8,045	49.5	0.539	−0.053
BO23	Unknown	CP018264.1	–	No	Unknown	0/5/0/0	11	6,323	51.0	0.546	0.022
K78	lp28-8	NZ_CP009066.1	Schüler et al. (2015)	No	Yes	2/0/0/0	11	6,092	51.7	0.551	0.021
PKo	Lp28-8, lp31	CP002947.1 CP000404.1	Casjens et al. (2011)	Partial	Unknown	2/0/0/0	8^b^	4,359	50.3	0.537	−0.006
***B. bavariensis***											
A104S	lp28-8	CP058821.1	Becker et al. (2020)	No	Unknown	1/0/0/0	6^b^	2,656	49.9	0.530	−0.107
PBaeII	Lp28-8	NZ_CP117803.1	Hepner et al. (2023)	Yes	Yes	1/0/0/0	8	4,600	49.5	0.545	−0.104
PBi	lp28-8	AY722928	Glöckner et al. (2004)	Partial	Unknown	3/0/0/0	10^b^	4,575	49.6	0.54	0.10
***B. maritima***											
CA690	lp36	CP044541.1	Margos et al. (2020b)	2 partial orfs	Unknown	N/A	10 cassette fragments	3,069 ^ic^	46.1	0.48	0.005
***B. mayonii***											
MN14-1420	lp28-10	CP015790.1	Kingry et al. (2016)	Yes	Unknown	1/14/0/0	24	14,648	48.5	0.412	−0.062
MN14-1539	lp28-10	CP015805.1	Kingry et al. (2016)	Yes	Unknown	1/9/0/0	17	10,786	48.5	0.411	−0.062
***B. spielmanii***											
A14S	lp28-8	CP001465.1	Schutzer et al. (2012)	Partial	Unknown	1/0/0/0	8^b^	4,627	52.5	0.520	0.052
***B. turdi***											
T1990A	Lp28-3	NZ_QBLN01000010.1	–	Yes	Yes	0/1/0/0	13	6,778	45.0	0.528	−0.031
***B. valaisiana***											
VS116	lp28-8	CP001442.1	Schutzer et al. (2012)	No	Unknown	1/0/0/0	3^b^	1,408	49.4	0.491	−0.055
***B. turcica***											
IST-7	lp35	CP028889.1	Gofton et al. (2018)	*vlp* sequences	Unknown	6/6/0/0	14	8,030	46.7	0.158	0.298

^aFS, frameshift; SC, stop codon. bIncomplete, more cassettes possible. cIn two regions. dEvidence for non-contiguous silent cassettes and vlsE. eSilent cassettes are telomere proximal.^

The identification of *vlsH* orthologs in *Borrelia* species and strains was performed using blastn or tblastn with other *vlsH* sequences, beginning with the *B. burgdorferi* B31 sequence. Many of the *vlsH* sequences are pseudogenes containing frameshifts, some of which are conserved in different strains (Supplementary Table S2). For comparative studies with relapsing fever *Borrelia vlp* and *vsp* sequences, complete ORF libraries were obtained from the NCBI database using a search of Assembly for the organism and strain name, retrieving the RefSeq page, and downloading the cds_from_genome file. Retrieval of *vlp* and *vsp* ORFs was based on annotation with those descriptors, limiting the searches to relatively complete and well-annotated genomes.

For the phylogenetic trees for VlsE amino acid sequences and *B. mayonii* strain silent cassette DNA sequences, MAFFT Version 7 (Katoh et al., 2019) in the UPGMA mode was utilized to construct the multiple sequence alignments and calculate Newick values. The online iTOL (Integrated Tree of Life) Program, Version 6.9.1^2^ was used for figure generation.

### 2.2 Nucleotide composition analysis

G + C percentage, GC skew (G-C)/(G + C), and AT skew (A-T)/(A + T) were obtained using data from the Emboss Explorer wordcount function^3^ or a Python script (available upon request). The coding strand was utilized for *vls* sequences and ORFs. For chromosome data, the approximate location of the origin of replication was determined as the cumulative GC skew minimum as calculated by ORI-Finder.^4^ The above parameters were then calculated for the leading strand of the left-and right-end portions of the chromosome; these two values were nearly equivalent and were thus averaged.

### 2.3 Protein comparisons and structural predictions

The alignment of VlsE protein sequences was performed using *Clustal Omega* followed by ESPript 3 processing of the sequence data (Sievers et al., 2011; Robert and Gouet, 2014). The locations of *α*-helices and *β*-pleated sheet secondary structures shown in Supplementary Figure S1 are based on the B31 VlsE crystal structure (Eicken et al., 2002). Structural predictions were performed using AlphaFold v2.3.0 (Jumper et al., 2021; Varadi et al., 2024) obtained from the source code at: https://github.com/deepmind/alphafold. Three-dimensional rendering, superposition, and Root Mean Square Deviation (RMSD) calculation were carried out using the PyMOL molecular graphics system and CCP4MG (McNicholas et al., 2011).

## 3 Results

### 3.1 Ubiquity of *vls* systems in Lyme borreliosis species and strains

*vls* sequences were found to be present in nearly all available complete genomic sequences from Lyme disease *Borrelia* species and strains, with exceptions being cases in which the *vls* locus-containing plasmid was apparently lost during *in vitro* passage. The regions containing *vls* sequences were identified by performing tblastn searches using the *B. burgdorferi* B31 allele VlsE1 predicted protein sequence (AAC45733.1) against the NCBI nucleotide database. Due to the large number of sequences from B31 vlsE variants in the database, sequences from this strain were excluded from the search. Using tblastn is advantageous because it identifies nucleotide regions that have similar predicted amino acid sequences, improving the identification of *vls* sequences despite the presence of both nucleotide sequence heterogeneity and frequent indels within the cassette regions.

**Figure 2:**
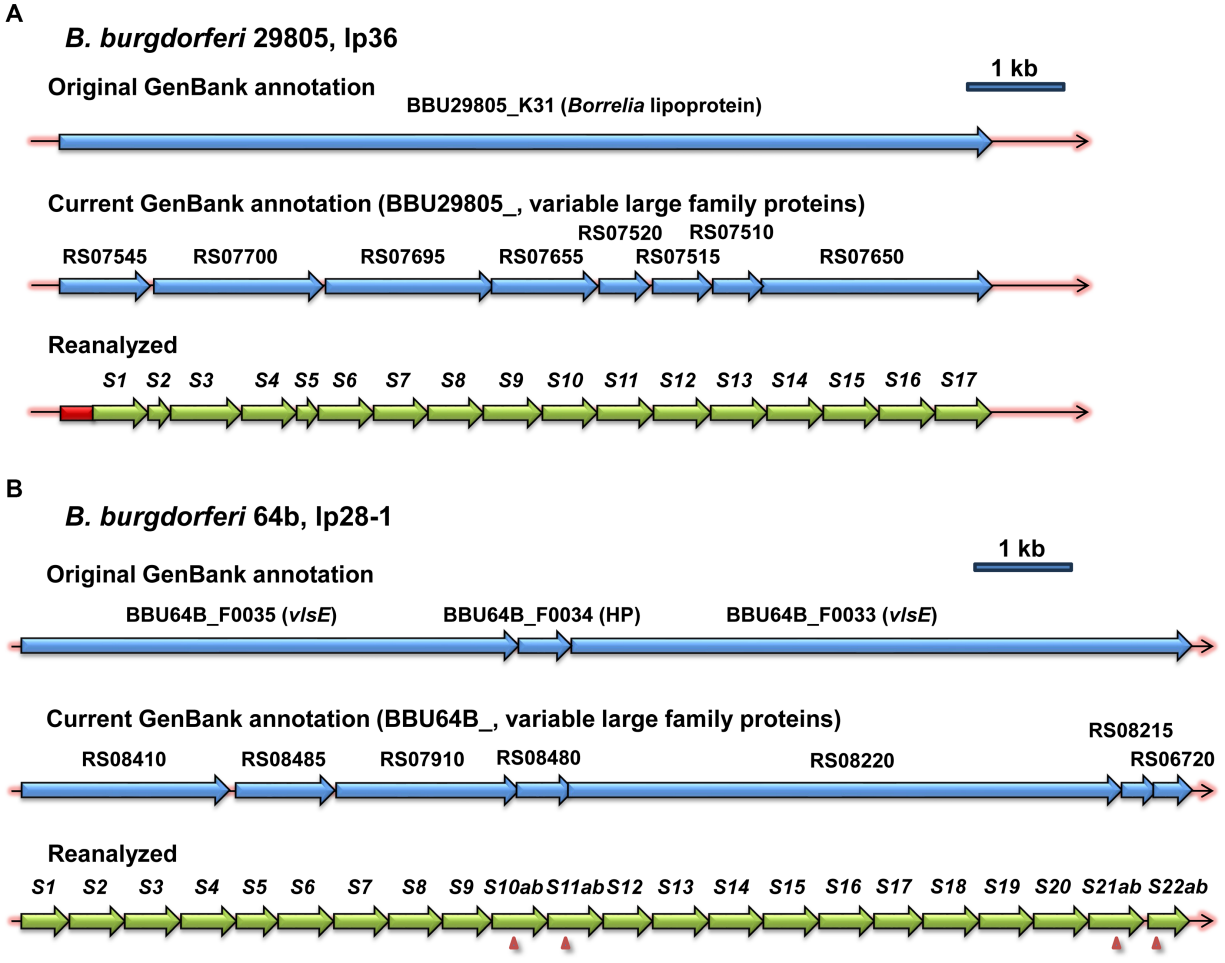
Examples of the inefficacy of automated annotation in the identification of *vls* sequences. The first two lines in each panel show the original and current annotations reported in the NCBI GenBank entries, whereas the third line depicts the annotation resulting from the reanalysis described in the Materials and Methods. *vlsS* silent cassettes are marked as *S1*, *S2*, etc. Frameshifts within silent cassettes are indicated by arrowheads, and the cassette segments on either side of the frameshift are given letter designations (e.g., *S10ab* = *S10a* and *S10b*). **(A)** *B. burgdorferi* strain 29805, lp36. **(B)** *B. burgdorferi* strain 64b, lp28-1.

*vls* silent cassette loci, which were identifiable due to the presence of multiple contiguous copies of *vls* cassette region sequences, were present in the available genomic sequences of 15 *B. burgdorferi*, 4 *B. afzelii*, 3 *B. garinii*, 3 *B. ba*var*iensis*, 2 *B. mayonii*, and 1 each *B. maritima*, *B. spielmanii*, *B. turdi*, and *B. valaisiana* strains (Table 1 and Supplementary Files 1, 2). Genomic sequences annotated using automated methods, most commonly the NCBI Prokaryotic Genome Annotation Pipeline (PGAP), typically had the corresponding ORFs annotated as encoding a large variable family protein, VlsE, a predicted lipoprotein, or a hypothetical protein (Figure 2). This outcome appears to be due to the limitation of automated protein sequence annotation to complete genes or conserved pseudogenes (with start codons) rather than protein-encoding gene segments such as the *vls* silent cassette loci. Thus simply searching GenBank file databases using keywords like “*vls*” is inadequate for identifying *vls* sequences. Some of the available Lyme disease *Borrelia* genomic sequences lack detectable *vls* sequences due to either limitation of submitted sequences to the chromosome or the apparent loss of *vls*-encoding plasmids in the clone used for sequencing.

The combined use of blast searches, automated and manual alignment with reference sequences (e.g., B31 *vlsE* or, if available, *vlsE* in the strain under analysis), and mapping of the sites using programs such as DNASTAR SeqBuilder was usually required for delineating the locations and start and end points of *vls* silent cassette (*vlsS*) sequences. BLAST protocols often truncate regions of sequence identity interrupted by variable regions, frameshifts, stop codons, or long indels. Because all of these are common in *vls* cassette regions, human discernment is required to identify elements such as single or multiple frameshifts within a *vlsS* sequence. Alignments with *vlsE* sequences aid in defining *vlsS* boundaries, as sequence identity with *vlsE* abruptly ends outside the cassette region. In general, the *vls* cassette boundary sequences are not well conserved among different LD *Borrelia* species and strains.

### 3.2 *vls* locus structure and annotation

**Figure 3:**
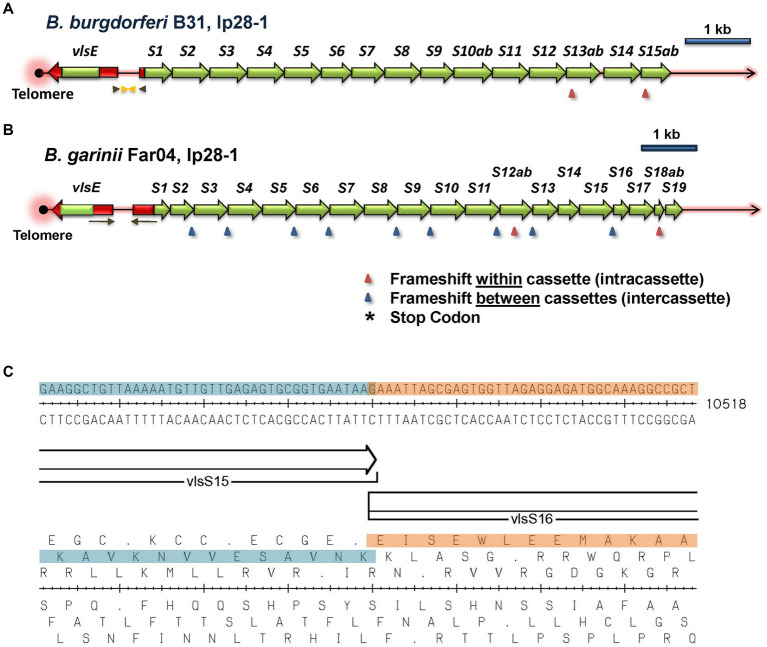
Overall architecture of typical *vls* loci. The *vlsE* expression site and the contiguous array of *vls* silent cassettes are arranged in a head-to-head configuration, with *vlsE* typically being located close to a telomere in the encoding linear plasmid. The silent cassettes represent complete or partial copies of the cassette region of *vlsE*, each with 6 variable regions (VRs; not shown). The first silent cassette is preceded by an in-frame gene segment with identity to the 5′ noncassette region of *vlsE* (shown in red), including the ribosome binding site but lacking the promoter region. Inverted repeats (arrows) of varying lengths and locations in each strain encompass parts of *vlsE*, the first silent cassette, and the intervening non-coding sequence. The silent cassettes represent a contiguous open reading frame interrupted by frameshifts and (less commonly) stop codons that may be located either within or between cassettes; the number and locations of these interruptions vary widely among different LD *Borrelia* strains, as shown in these two examples. In this study, the silent cassettes are numbered sequentially beginning with *vlsS1*; cassette segments subdivided by reading frame interruptions are designated by letters, e.g., *vlsS13a* and *vlsS13b*. **(A)** *B. burgdorferi* strain B31, lp28-1. **(B)** *B. garinii* strain Far04, lp28-1. **(C)** An example of an intercassette frameshift occurring between *vlsS15* and *vlsS16* of *B. garinii* Far04. The DNA sequence, the two silent cassettes with a 1 bp overlap, and the six reading frames are shown.

Examples of intact *vls* locus structures of Lyme disease *Borreliae* are provided in Figure 3, with additional information available in Supplementary Files 1, 2. The *B. burgdorferi* B31 locus (Figure 3A) consists of an expression site called *vlsE*, an intervening noncoding region, a segment that is identical to the 5’end of *vlsE*, and a contiguous series of silent cassettes that resemble the central cassette region of *vlsE*. *vlsE* and the *vls* silent cassette array are in the opposite orientation. The silent cassettes, which are defined by their sequence homology to the central cassette region of *vlsE*, often form long, contiguous open reading frames encompassing several adjacent silent cassettes. In some organisms such as *B. garinii* Far04 (Figure 3B), intercassette (between cassette) frameshifts are common in the *vls* cassette region. The intercassette frameshifts often have a common structure within each strain, such as the one bp overlap between neighboring silent cassettes *vlsS15* and *vlsS16* shown in Figure 3C. Intracassette (within cassette) frameshifts and stop codons are also present with variable locations and frequency.

Most of the available genomic sequences lack *vlsE*, likely because of its proximity to the telomere and the presence of an inverted repeat between *vlsE* and the *vls* silent cassette array. Both of these features greatly reduce cloning efficiency. As a result, *vlsE* and surrounding sequences were missing from nearly all of the genomic sequencing studies that utilized cloning of DNA segments into a recombinant plasmid followed by Sanger sequencing. Illumina sequencing of uncloned, fragmented DNA appears to provide more efficient analysis and assembly of the telomere, *vlsE*, and its upstream region.

In all 10 of the intact *vls* loci, *vlsE* and silent cassettes are arranged in a head-to-head configuration, with intervening intergenic regions of 334, 377, 298, 377, 334, 375, 479, 436, 436, and 483 base pairs, respectively, for *B. burgdorferi* strains B31, B17/2013, JD1, MM1, and PAbe, *B. garinii* Far04, *B. ba*var*iensis* PBaeII, *B. mayonii* strains MN14-1420 and MN14-1539, and *B. turdi* T1990A. For all strains, the silent cassette region starts with a copy of the 5′ region of the *vlsE* reading frame, although for B31 and PAbe this region is truncated on the 3′ end. For *B. garinii* Far04 and the two *B. mayonii* sequences, the sequence identity starts with the ribosome binding site (RBS) and start codon. In JD1, the RBS and the first two codons of the coding region are missing. Sequence identity extends through the *vlsE*-like 5′ end into the cassette region, and stops at the first site of sequence variation between *vlsE* and silent cassette 1. None of these silent cassette loci contain a recognizable promoter region. Inverted repeat sequences are present in the shared 5′ sequences as well as portions of the intervening noncoding region; however, the sequence identity does not extend into the *vlsE* promoter region, in that the silent cassette array lacks a promoter. There is a variable number of silent cassettes in each strain, most likely because of the occurrence of duplications, recombinations, and deletions, as described below. Many available GenBank entries also contain incomplete sequences of the *vls* locus, missing silent cassettes and other elements. However, more recent studies have utilized more efficient sequencing techniques, often using both long-and short-read technologies (Schüler et al., 2015; Kingry et al., 2016; Gofton et al., 2018; Becker et al., 2020; Margos et al., 2020b), improving the completeness and accuracy of the available sequences.

As described previously (Zhang et al., 1997; Norris, 2014; Chaconas et al., 2020), the *vls* cassette region exhibits distinctive properties, including a high overall G + C content and a pronounced GC skew on the coding strand (Table 1; Supplementary Figure S2). These combined properties are unique to the *vls* cassettes, and are not present in the RF *Borrelia vlp/vsp* antigenic variation system or in the *vlsH* gene of LD *Borrelia* (Supplementary Figure S2); *vlsH* is a LD *Borrelia* homolog of *vlsE* that is located on a different plasmid and does not undergo sequence variation (Supplementary Table S2) (Norris, 2014). As previously described (Walia and Chaconas, 2013; Chaconas et al., 2020), a high proportion of G’s on the coding strand are clustered in groups of 3 to 5, an arrangement commonly found in G-quadruplex structures (G4-S). The combination of high G + C, GC skew, and the clustering of G’s on the coding strand is thus likely to be important in *vlsE* sequence variation. In *B. burgdorferi* B31, a 17 bp direct repeat sequence is found at both ends of the *vlsE* cassette region. However, in other species and strains, the two ends of the *vlsE* cassette region generally are not the same (Supplementary Figure S1) (Wang et al., 2003; Walia and Chaconas, 2013; Norris, 2014; Chaconas et al., 2020). All of the *vls* loci have six prominent variable regions (VRs), in which the sequence differences between the silent cassettes are concentrated. The positions of these VRs within the cassettes can differ to some extent between strains.

In this article, the *vls* loci are depicted with *vlsE* on the left end, in that this orientation provides a clearer view of the silent cassette array structure; thus the sequences represent the reverse complement relative to most genomic sequences. In addition, the silent cassettes are numbered consecutively as *vlsS1*, *vlsS2*, and so forth. For consistency, the original designations of *vls2* through *vls16* for the silent cassettes of *B. burgdorferi* B31 have been replaced by *vlsS1* through *vlsS15* in this article. Cassette segments in which the reading frame is interrupted by a frameshift or stop codon are designated by an added letter, e.g., *vlsS10a* and *vlsS10b* in Figure 2A. The *vlsS* designation provides a better distinction between the silent cassettes (which are invariant in a given strain, except for long time-scale evolutionary changes) and the *vlsE* central cassette (which undergoes frequent variation during mammalian infection).

### 3.3 Evidence for evolution in the *vls* locus

The overall arrangement of the *vls* locus is highly conserved among the Lyme disease *Borrelia*. Also, within each strain, the *vls* region sequences are by in large non-variant, except for the changes in *vlsE* occurring as a result of the antigenic variation process. In contrast, the *vls* sequences of the different species and strains have diverged substantially from one another, making it challenging to discern how evolution in this locus occurs. However, this meta-analysis uncovered rare instances in which pairs of strains have an overall high degree of sequence identity and synteny with occasional genetic events, permitting examination of the nature of changes resulting in divergence. The types of events observed include: (1) point mutations and short insertion/deletion or recombination events; (2) sizeable duplication events; and (3) presence of multiple duplication and rearrangement events, as described below.

### 3.4 Occurrence of short regions of heterogeneity of the *vls* loci between *B. burgdorferi* strains B31 and PAbe

*B. burgdorferi* B31 was the first LD *Borrelia* strain isolated and characterized, and was derived from *I. scapularis* specimens from Shelter Island, New York, United States (Burgdorfer et al., 1982; Schwan et al., 1988). A surprising observation is that multiple strains isolated from patients in Germany (strains Pref1, PBre, PKa2, PAbe, and PBoe) have closely related *vlsE* sequences to those of B31, leading to their description as “B31-like strains.” As a measure of the close relatedness of this group of strains, the chromosomes of strains PAbe and B31 differ at only 81 of 910,728 base pairs (0.009%), as compared 3,395 of 910,728 base pairs (0.39%) between PAbe and the more divergent *B. burgdorferi* strain MM1. PAbe is the only one of these German strains that for which lp28-1 plasmid sequence with a *vls* locus is available. This plasmid is nearly identical to lp28-1 of B31, with the only differences being within *vlsE* and the first four silent cassettes of the *vls* locus. The other B31-like strain (PAli) for which a genome sequence is available is lacking lp28-1, most likely because the plasmid was lost during *in vitro* culture prior to genomic sequencing.

**Figure 4:**
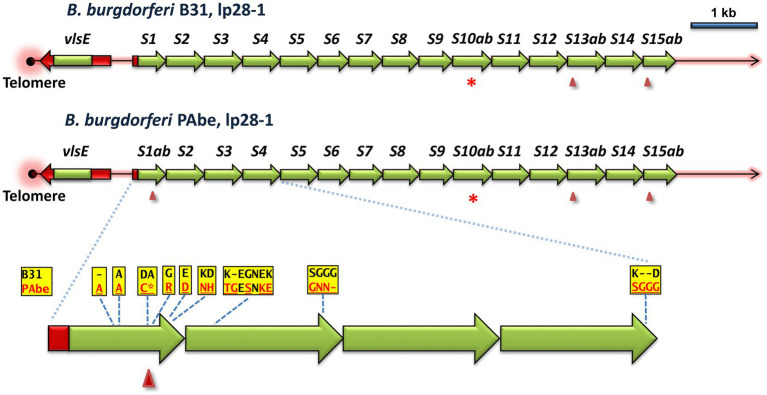
Comparison of the *vls* regions of the closely related *B. burgdorferi* strains B31 and PAbe reveals their divergence through point mutations and short indels. The silent cassette regions are identical except for the differences shown in the inset; the divergent amino acid sequences resulting from the nucleotide differences are shown. Loss of amino acid codons due to either 3-or 6-bp indels are indicated by dashes. A frameshift at the end of *vlsS1a* (marked by an arrowhead) results in a stop codon (asterisk); *vlsS1b* begins at the same location in another reading frame.

The differences in the silent cassettes of the B31 and PAbe strains consist of isolated point mutations or short regions of heterogeneity within the cassettes *vlsS1* through *vlsS4* (Figure 4). These represent SNVs or short, 3-or 6-base indels (30 nt total) resulting in 18 encoded amino acid differences and one frame shift. The B31 and PAbe silent cassette regions are otherwise identical in sequence. It is not known why the differences are clustered in a relatively short region of the silent cassettes. However, it is unlikely that they are due to sequence errors, in that the B31 silent cassette region has been sequenced multiple times with identical results (Fraser et al., 1997; Zhang et al., 1997; Casjens et al., 2000; Combs et al., 2022) and the PAbe genome was sequenced using both short read (Illumina) and long read (PacBio) methods (GenBank entry NZ_CP019923.1). Therefore the sequence differences are likely the result of mutations that occurred during the divergence of these two closely related strains.

### 3.5 Duplication events in *B. garinii* Far04 and *B. burgdorferi* 64b silent cassettes

**Figure 5:**
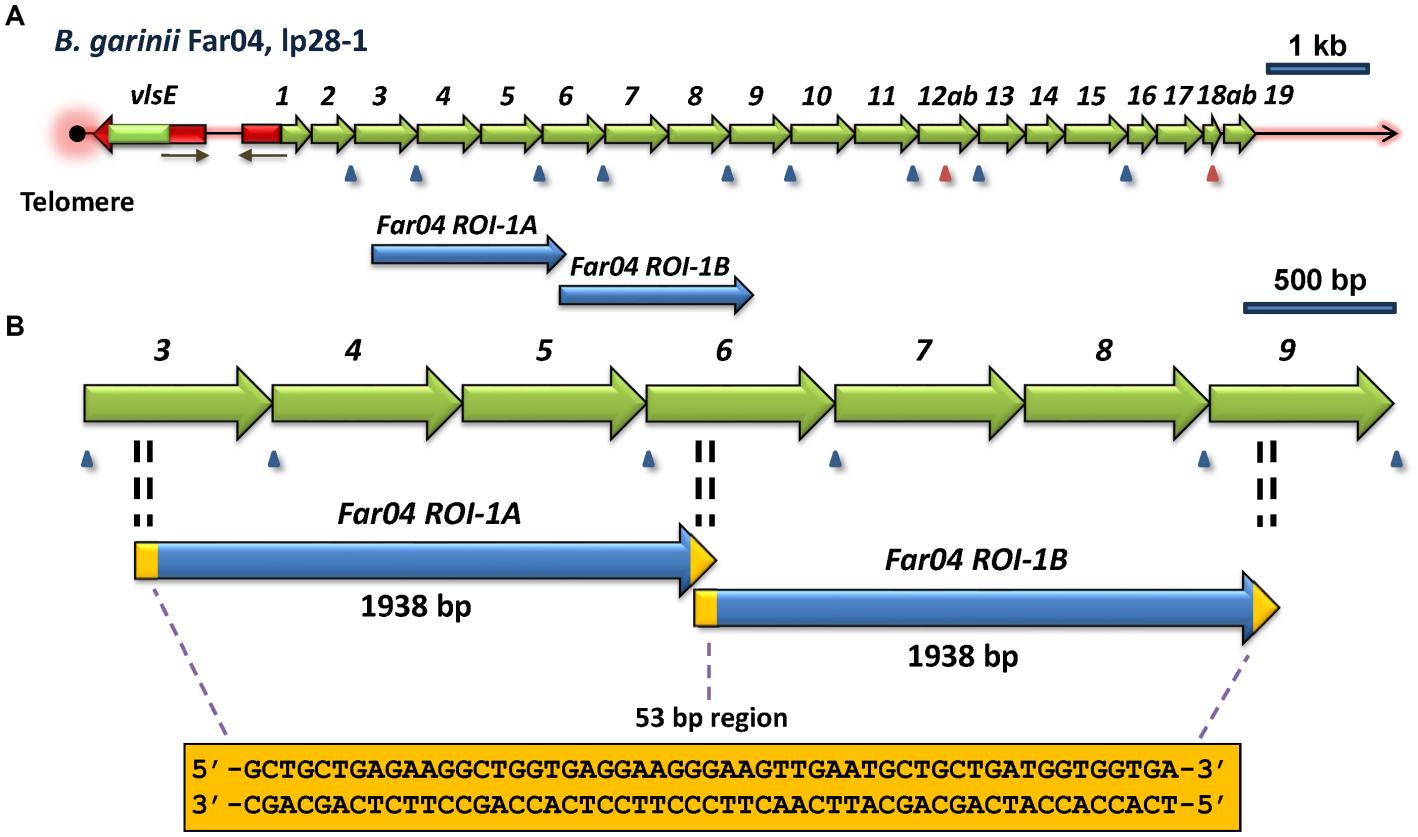
Regions of identity (*ROI*) indicative of a duplication event within the *vls* locus of *B. afzelii* Far04. **(A)** Overview of the *vls* locus, showing the location of the two *ROI*’s. **(B)** Enlargement of the area containing Far04 *ROI-1A* and Far04 *ROI-1B*, 1,938 bp identical sequences that overlap by 53 bp. The sequence of the 53-bp overlap region is found at both ends of each *ROI*, consistent with a duplication due to a crossover between two copies of the plasmid within the 53-bp region. The 3′ end of *vlsS3*, all of *vlsS4* and *vlsS5*, and the 5′ region of *vlsS6* are identical to the 3′ end of *vlsS6,* all of *vlsS7 and vlsS8*, and the 5′ end of *vls9*, respectively.

One of the most common mechanisms of genetic change is a duplication event, resulting in a direct or inverted repeat of a nucleotide sequence. In the search for intragenic sequence similarities, we identified a direct repeat of *vls* silent cassette sequences in the *B. garinii* Far04 lp28-1 sequence (Figure 5). These identical repeat sequences, identified as Far04 Regions of Identity (*ROI*) *Far04 ROI-1A* and *Far04 ROI-1B*, each consist of 1,938 bp. *Far04 ROI-1A* encompasses the 3′ portion of *vlsS3* to a 5′ segment of *vlsS6*, whereas *Far04 ROI-1B* consists of a 3’region of *vlsS6* to a 5′ region of *vlsS9* (Figure 5A). There is a 53-bp overlap between these regions of identity. The most likely genetic scenario is a homologous recombination crossover event occurring between two sister copies of lp28-1 within the 53-bp region located at both ends of the original 1,938 bp nucleotide segment (Figure 5B). Regardless of where the crossover occurs within the 53-bp repeat, the result would be the same. This chimeric form of lp28-1 would undergo replication and in the Far04 strain became the sole copy of this plasmid. Another possibility is a single plasmid slipped strand synthesis event in which the replicating strand re-annealed a second time to the initial 53-bp region; however, this scenario seems less likely given the 1,938 bp distance involved.

Two duplication events were also observed in the *B. burgdorferi* strain 64b (Supplementary Figure S3). One pair of identical *ROI* sequences are 1,114 bp in length, whereas the other pair are 471 bp. Unlike the Far04 duplication event, each pair of the 64b ROI sequences are separated from one another and are surrounded by dissimilar sequences. Therefore, the potential genetic mechanisms leading to these duplication events are less clear.

### 3.6 Complex evolution of the vls locus in *B. mayonii* strains MN14-1539 and MN14-1420

*B. mayonii* strains MN14-1539 and MN14-1420 (hereafter called 1539 and 1420) have closely related *vls* systems (Kingry et al., 2016). The genomic sequences of the *vls* systems in both strains are complete, containing intact *vlsE* genes and *vlsS* silent cassette arrays. Strain 1539 has 17 silent cassettes, whereas strain 1420 has an expanded set of 24 cassettes. Many of the *vls* silent cassettes in the two strains have a high degree of sequence homology, as indicated in a phylogenetic tree constructed using alignments of these silent cassettes (Supplementary Figure S4). It was noted that 9 of 17 cassettes in strain 1539 having 100% nucleotide identity with cassettes in strain 1420; an additional 6 cassettes have >98% nucleotide identity with strain 1420 cassettes. Another novel aspect of the strain 1539 and 1420 *vls* loci is the concordance of cassette order, which has otherwise only been found in the *B. burgdorferi* strains B31 and PAbe (Figure 4). The high degree of similarity in the *vls* systems of these two strains indicates a close evolutionary relationship between them, providing an opportunity to gain insight into the types of genetic events that contribute to the generation of this remarkable antigenic variation system.

**Figure 6:**
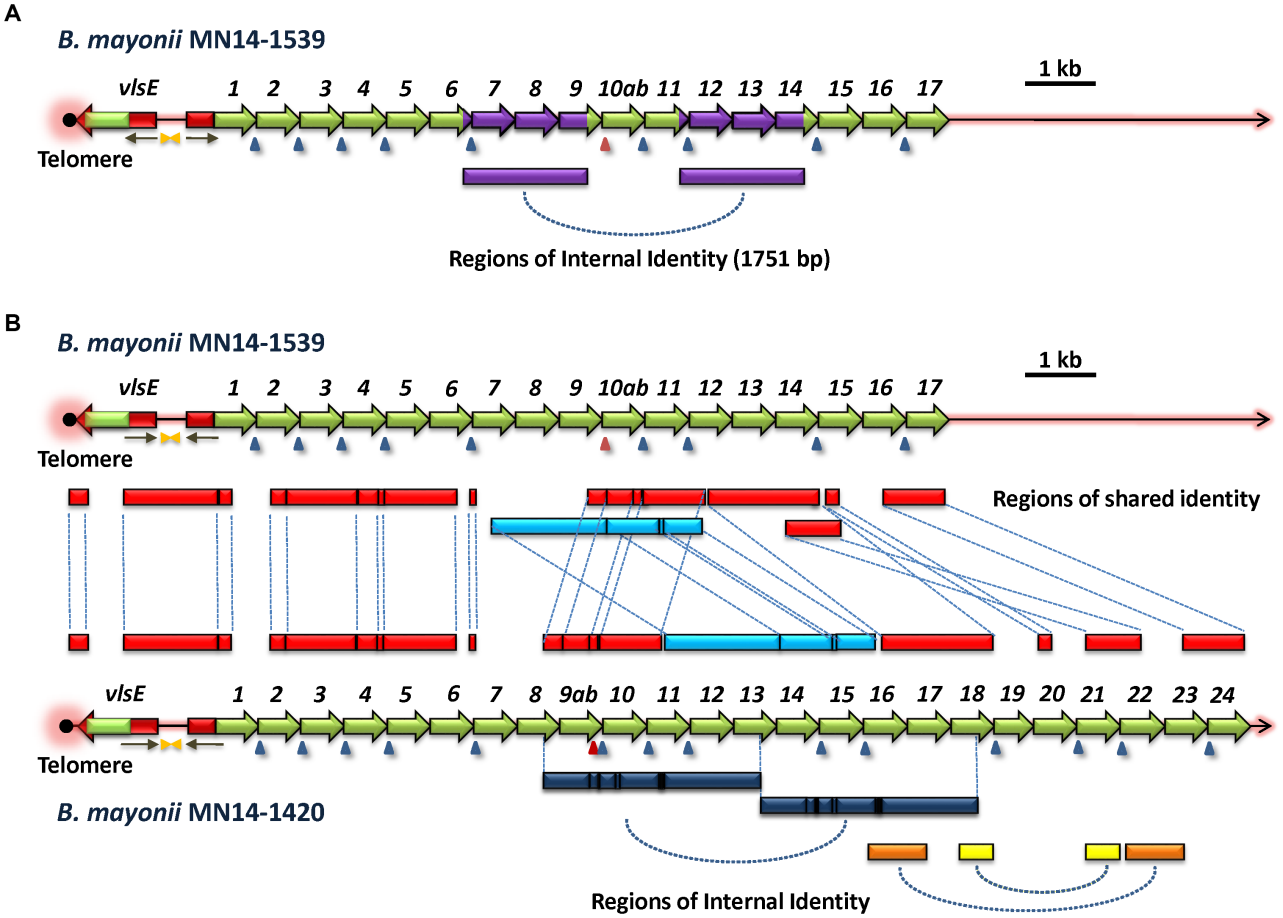
Common ancestry and divergence of the *vls* loci of *B. mayonii* strains MN14-1539 and MN14-1420. **(A)** *B. mayonii* MN14-1539 contains two identical 1,751-bp regions (purple), indicating an intrastrain duplication event. **(B)** Comparison of the two *vls* loci reveals the presence of multiple shared regions of identity (RoSI) between the two strains (red). An apparent translocation rearrangement has shifted the location of one region of identity (light blue) that has also diverged through point mutations and small indels (black lines). The *B. mayonii* MN14-1420 *vls* silent cassette region has expanded relative to that of MN14-1539 due in part to apparent duplication events, as indicated by intrastrain regions of identity (dark blue, yellow, and orange bars at the bottom of the diagram).

As with *B. garinii* Far04, *B. mayonii* 1539 and 1420 were each found to have large internal regions of sequence identity (Figure 6). Strain 1539 has two identical regions (*1539 RoSI-1A* and *1539 RoSI-1B*) each comprising 1,751 bp. Unlike what was observed in Far04, the regions of identity in strain 1539 are not contiguous, but are separated by 1,316 bp. Like strain 1539, *B. mayonii* strain 1420 also has large duplicated regions of sequence similarity (Figure 6B, lower panel). The lengths of these two regions are 3,064 bp and 3,072 bp, respectively. In this case, the two regions are punctuated by short segments of sequence differences, consisting of indels or nucleotide substitutions. Overall, the aligned sequences of these two regions in the strain 1420 *vlsS* cassettes are identical at 3,050 of 3,076 positions, representing 99.15% identity. This degree of identity is much greater than that between the pairings of other *vls* sequence regions (~94%), indicating that the regions were first duplicated and underwent diversification through point mutations or short recombination events. Based on these observations, it is likely that *vls* region duplication events occurred in an ancestral *B. mayonii* precursor of both strains 1539 and 1420, and these regions have since diverged but still retain considerable sequence identity.

Global comparison of the *vls* system sequences of *B. mayonii* strains 1539 and 1420 revealed extensive sequence identity as well as rearrangements and other forms of divergence (Figure 6B). In this depiction, all boxes between the two sequence maps represent regions of sequence identity between the two strains, with dashed lines linking the corresponding regions. The telomere, *vlsE* gene, and upstream region (including inverted repeats and the 5′ *vlsE*-like sequence associated with *vlsS1*) were all highly conserved, with the expected divergence in the *vlsE* cassette region due to antigenic variation. With the exception of a divergent region at the intersection of *vlsS1* and *vlsS2*, the sequences and order of *vlsS1* through *vlsS6* were nearly identical. As mentioned previously, this preservation of recognizable silent cassette order is rarely observed in comparisons of other strains. The first major rearrangement is evident after *vlsS6*, with an apparent insertion of sequences corresponding to parts of *vlsS7* and *vlsS8* in strain 1420. These sequences do not show close homology to other silent cassette regions in the two strains. A major region (2,979 bp, marked in light blue in Figure 6B) has undergone a relocation, changing its location to a position after another shared region (in red) in strain 1420. Additional regions of identity are located further downstream, punctuated by regions of non-identity representing apparent insertions of *vls* sequence segments in strain 1420. Overall, this analysis is consistent with the *vls* systems of *B. mayonii* strains 1539 and 1420 arising from a common ancestor strain and then diverging through large insertions, rearrangements, and introduction of smaller regions of sequence differences through localized recombinations, indels, and point mutations.

### 3.7 The cryptic *vls* region of *B. maritima*

**Figure 7:**
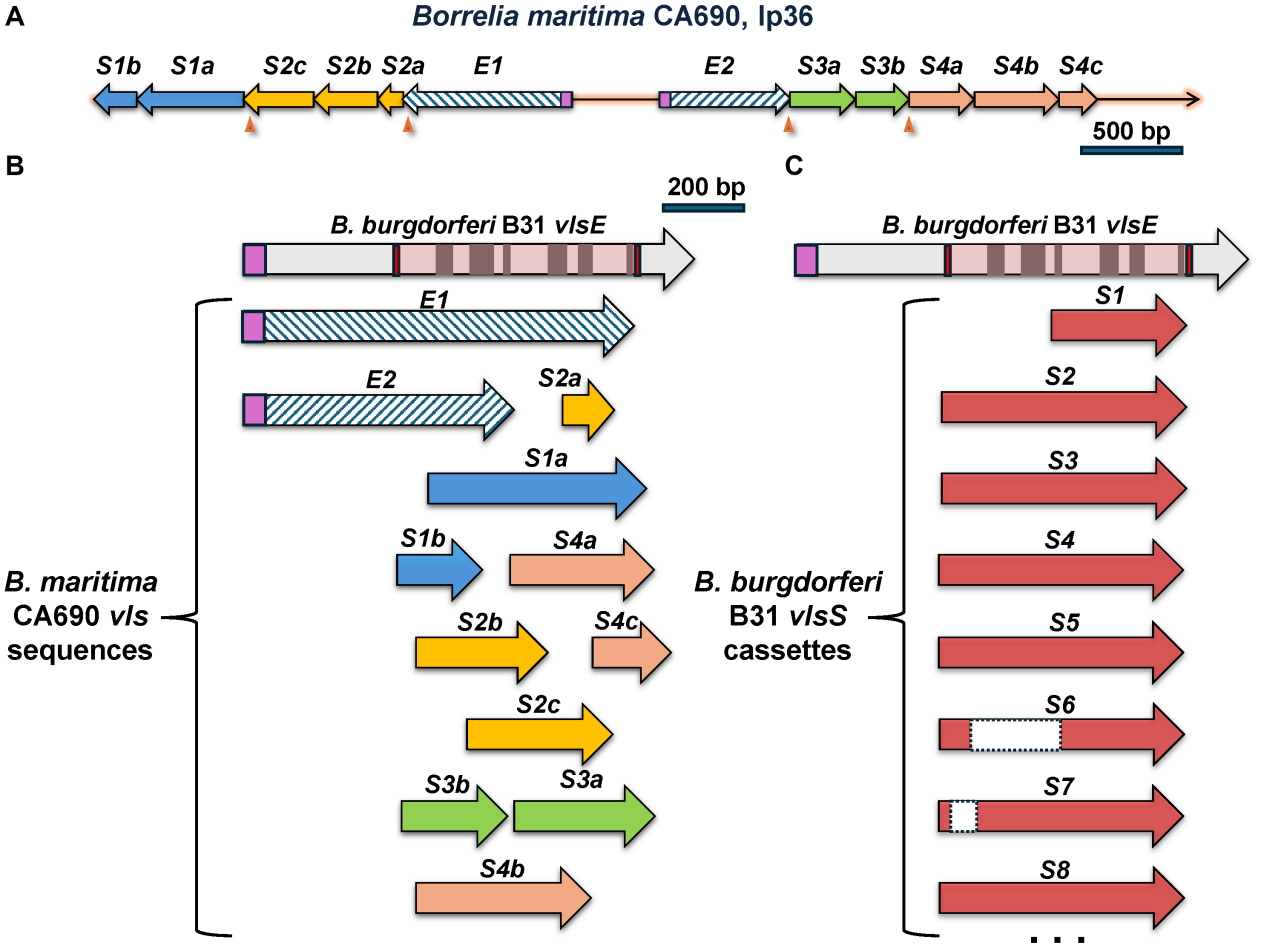
The unusual *vls* system of *B. maritima* CA0690. **(A)** Overall arrangement of the region containing *vls* sequences. These include *vlsEORF1* (abbreviated as *E1*) and *vlsEORF2* (*E2*), which encode the truncated *vlsE* coding sequences indicated by hatched arrows. Signal peptidase II leader sequences are shown in purple. Additional ORFs, *vlsSORF1* through *vlsSORF4*, are separated from the *vlsEORF*s and each other by frameshifts (arrowheads). The *vlsSORF*s are further subdivided into regions encoding different portions of the cassette region reading frame, e.g., *vlsSORF1a* (*S1a*) and *vlsSORF1b* (*S1b*). **(B)** Alignment of the *B. maritima vls* sequences with *B. burgdorferi vlsE* shows their jumbled, disorganized nature. *vlsEORF1* and *vlsEORF2* encode incomplete portions of the *vlsE* reading frame. The *B. maritima vlsS* segments align with varied lengths and locations of the cassette region; as an example, the *vlsSORF3a* and *vlsSORF3b* segments encompass two regions of the cassette region in the opposite order of their genomic locations. This disordered arrangement contrasts with the usually full-length silent cassettes (with some truncations and internal deletions) in the *B. burgdorferi* B31 silent cassettes, as shown in **(C)**.

An exception to the canonical *vlsE*-*vlsS* cassette arrangement was found in the LD group spirochete *B. maritima* strain CA690, isolated from an *Ixodes spinipalpis* nymph in Northern California (Margos et al., 2020b). The CA690 lp36 sequence contains the locus depicted in Figure 7, which contains two contiguous stretches of *vls*-related sequences oriented in opposite directions. The first ORF in each of these stretches (here called *vlsEORF1* and *vlsEORF2*) encodes amino acid sequences homologous to VlsE, but both are lacking the sequences encoding the C-terminal portion of the protein in comparison with the B31 *vlsE* (Figure 7B). Although each of these partial *vlsE* homologs have a predicted ribosome binding site, leader sequence, and *vlsE*-like 5′ region, they do not have recognizable promoter sequences and thus likely represent pseudogenes; this same pattern is typical of *vlsS1* in other LD *Borrelia*. Both *vlsEORF1* and *vlsEORF2* end with frameshifts and are followed by four ORFs (*vlsSORF1 – vlsSORF4*) encoding *vls* sequences (Figure 7A). Rather than encoding intact silent cassettes, these downstream ORFs encode jumbled fragments corresponding to different portions of the cassette region (Figure 7B). This arrangement contrasts with the usual orderly array of complete silent cassettes with some truncations and indels that occur in other LD *Borrelia*, as exemplified by the B31 system (Figure 7C). These findings suggest that the *B. maritima* CA690 *vls* region is in transition, either in the early stages of formation or the late phase of decay. The possibility that this arrangement is due to sequence mis-assembly is effectively negated by the use of both short read (Illumina) and long read (Oxford Nanopore) data in the genome assembly (Margos et al., 2020b).

### 3.8 The *Borrelia turcica vlp* system – an example of convergent evolution?

**Figure 8:**
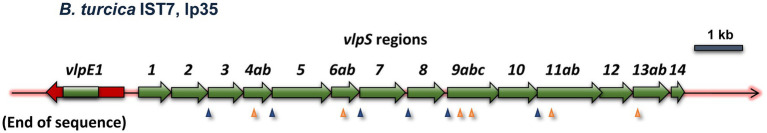
The variable large protein (*vlp*) system of the relapsing fever family organism *B. turcica* IST7 appears to exemplify convergent evolution toward a system resembling the antigenic variation system of Lyme disease *Borrelia*. The *vlpE* and *vlpS* regions of *B. turcica* IST7 are clearly more homologous to *vlp* sequences found in other relapsing fever *Borrelia* (see text). However, the cassette region nature of the *vlpS* gene segments, their near-contiguous arrangement, and the head-to-head arrangement of *vlpE* and the *vlpS* all more closely resemble the LD *Borrelia vls* system than the typical *vlp/vsp* systems of relapsing fever *Borrelia*. Frameshifts are indicated with arrowheads, as described in Figure 2.

*B. turcica* IST7 is a relapsing fever group *Borrelia* organism isolated from a *Hyalomma aegyptium* tick feeding on a tortoise in Turkey (Gofton et al., 2018). It is related to “*Borrelia tachyglossi* candidatus,” identified in the blood of an echidna (Loh et al., 2017; Gofton et al., 2018). While these two organisms most closely align with the genetic content of relapsing fever *Borrelia*, they also share some characteristics with Lyme disease spirochetes (Gofton et al., 2018). The *B. turcica* IST7 linear plasmid lp35 has an open reading frame encoding a 380-aa predicted lipoprotein characterized as a member of the variable large protein family by RefSeq, which includes both VlsE and Vlp homologs. This predicted protein (here called VlpE1) has a significant but relatively low degree of homology to relapsing fever Vlp proteins (e.g., 32% identity, 46% similarity to *Borrelia coriaceae* Vlp WP_025408854.1). A phylogenetic tree constructed with representative Vlp and VlsE proteins indicates that *B. turcica* IST7 VlpE1 is more closely to relapsing fever Vlp sequences than to LD VlsE sequences (Supplementary Figure S5). Adjacent to the telomere-localized *vlpE1* gene, there is a nearly contiguous series of nucleotide sequences in the opposite orientation representing variants of the central region of *vlpE1* (Figure 8). This overall arrangement is highly similar to the configuration of Lyme disease *vls* systems, although the size of the regions covered by these *vlp* “cassettes” is more variable than those found in *vls* silent cassettes. No other *vlp* sequences are found in the remainder of the *B. turcica* genome, and only one variable small protein (*vsp*) encoding gene (also located on lp35) is present. This arrangement is unlike that found in the *vlp/vsp* systems of any other relapsing fever *Borrelia* characterized thus far, in that the *vlp* and *vsp* gene segments are typically scattered throughout one or two linear plasmids and are often in opposite orientations. In addition, these gene segments represent nearly the full length of *vlp* or *vsp* genes, missing only the promoter and first few amino acid codons present in the variable membrane protein (Vmp) expression site. Thus in many ways the *B. turcica vlp* system more closely resembles the *vls* system of Lyme disease *Borrelia* than the antigenic variation system found in relapsing fever *Borrelia*.

### 3.9 Structural predictions indicate a high degree of conservation of VlsE structure despite framework amino acid divergence

Alignment of VlsE predicted amino acid sequences obtained from 5 LD *Borrelia* species and 19 strains demonstrate the presence of highly conserved regions but also regions of considerable diversity (Supplementary Figure S1). As expected, some of these diverse regions correspond to the locations of VR1 through VR6. These VRs undergo extensive sequence variation during mammalian infection; thus the sequence obtained in any one clone of a strain is just a “snapshot” of the highly variable sequences in each VR. However, there are other regions that represent heterogeneity between the different species and strains that represent “framework” sequence divergence that is not due to the antigenic variation mechanism. This heterogeneity is illustrated in a phylogenetic tree format in Supplementary Figure S5. This representation shows, for example, the close grouping of the “B31-like” strains and also the early divergence of VlsE from the relapsing fever Vlp sequences.

**Figure 9:**
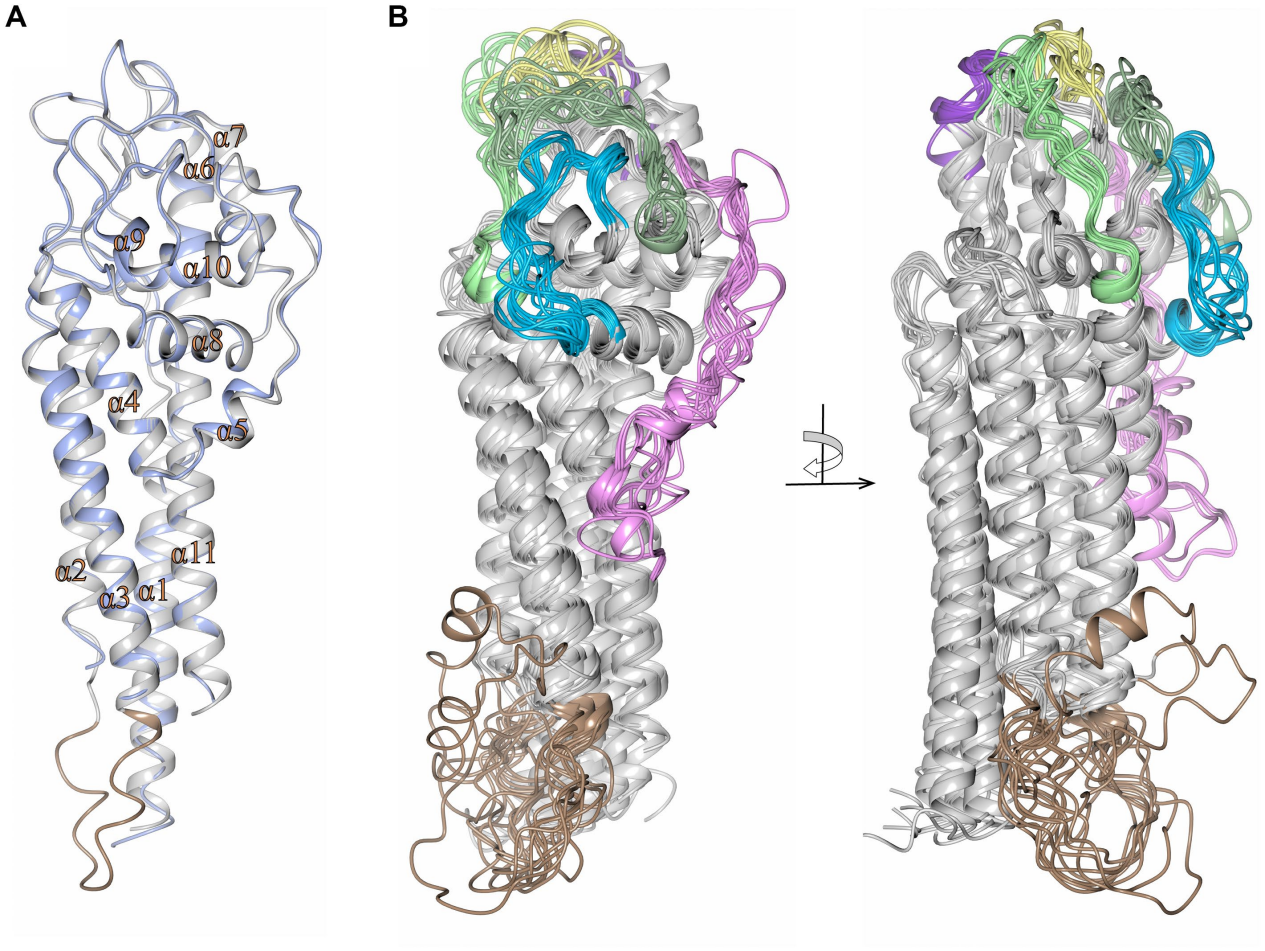
High concordance of the predicted structures of VlsE proteins from 19 LD *Borrelia* organisms. **(A)** Crystal structure of VlsE from *B. burgdorferi* B31 (PDB ID 1L8W, blue) superimposed with the AlphaFold predicted structure covering residues 29–346 (gray; RMSD 0.67 Å). All 11 *α*-helices in the crystal structure have been designated as α1 through α11. The region of heterogeneity between α2 and α3 is illustrated in light brown. **(B)** Superimposed AlphaFold predicted structures of VlsE from *B. burgdorferi* B31 with *B. burgdorferi* 297 (RMSD 2.01 Å), *B. burgdorferi* JD1 (RMSD 1.39 Å), *B. burgdorferi* PBoe (RMSD 0.78 Å), *B. burgdorferi* PAbe (RMSD 0.66 Å), *B. burgdorferi* PBre (RMSD 0.84 Å), *B. burgdorferi* PKa2 (RMSD 0.75 Å), *B. burgdorferi* PRef1 (RMSD 0.89 Å), *B. afzelii* PKo (RMSD 2.12 Å), *B. garinii* Pli (RMSD 1.58 Å), *B. garinii* Far04 (RMSD 1.40 Å), *B. garinii* PBr (RMSD 1.95 Å), *B. mayonii* 1539 (RMSD 1.62 Å), *B. mayonii* 1420 (RMSD 1.57 Å), *B. finlandensis* SV1 (RMSD 1.46 Å), *B. spielmanii* PMew (RMSD 2.01 Å), *B. spielmanii* A14S (RMSD 1.74 Å), *B. spielmanii* PHap (RMSD 2.22 Å), and *B. turdi* T1990 (RMSD 1.48 Å). The structures are shown at two angles rotated by 90 degrees. The variable regions (VRs) are illustrated with light green (VR1), pink (VR2), yellow (VR3), blue (VR4), dark green (VR5), and dark purple (VR6) colorations. The light brown coloration at the membrane proximal portion of the VlsE proteins corresponds to the heterogeneous region represented by positions 94 to 112 in the aligned sequences in Supplementary Figure S1.

It has been challenging to obtain high quality crystals of VlsE for 3D structural determinations. To date, the only crystal structure of VlsE that has been obtained (Eicken et al., 2002) is from a single variant (called VlsE1) from the strain *B. burgdorferi* B31. To examine whether the framework heterogeneities observed in the alignment in Supplementary Figure S1 give rise to significant structural differences among the different species and strains, the program AlphaFold was used to predict the 3D structures for each of the 19 different VlsE sequences (Figure 9). We first demonstrated that the AlphaFold predicted structure of VlsE1 closely resembled the previously obtained crystal structure (Figure 9A). The superimposed predicted structures of the 19 VlsE proteins also exhibited a high degree of concordance, particularly with regard to the major *α* helices that comprise the core framework of VlsE (Figure 9B). As expected, the six VRs located near the membrane distal surface of the VlsE proteins exhibited variance in terms of their predicted structures, although not as much as one might expect given the high degree of amino acid insertion, deletion, and substitution observed in these regions. The overall structure of the proteins is not affected by these differences, presumably because these VRs are located in loop regions. The confidence score, known as the predicted local distance difference test (pLDDT), for the loop regions harboring the six VRs for the most part showed the value above 70, indicating a generally good backbone prediction. A relatively lower pLDDT value was observed for the VR2 region located between helices α4 and α6 and includes the short α-helix 5 (colored pink in Supplementary Figure S1 and Figure 9B). The sequence differences (due to both VR variation and species and strain heterogeneity) are reflected in different electrostatic potential surface patterns where again the distal surface of the VlsE proteins harboring the VRs show significant variation between the members (Supplementary Figure S6). Areas of relative heterogeneity of non-VR “framework” regions of VlsE (e.g., the C-terminal region in Supplementary Figure S1) retained the corresponding structural elements observed in VlsE1. Another heterogenous region which includes residues 94–112 covering the loop region between helices α2 and α3 (the light brown colored box in Supplementary Figure S1) was not resolved in the previously determined crystal structure of *B. burgdorferi* B31 VlsE1 (PDB ID 1L8W) (Eicken et al., 2002), while in the AlphaFold predicted structure, the average pLDDT value of this region was 48, strongly indicating the flexible nature of the corresponding region (Figure 9A). According to the pLDDT plot generated by ColabFold (v1.5.5) (Mirdita et al., 2022), this region stands out as having one the lowest pLDDT values in all predicted protein structures (Supplementary Figure S6). Therefore, it is not surprising that this region (colored light brown in Figure 9) exhibited a low degree of concordance. In sum, these results indicate that VlsE proteins expressed by a wide variety of LD *Borrelia* species and strains are likely to have a very similar overall structures, bearing a constant framework for the “display” of the frequently changing VRs on the membrane distal surface.

**Figure 10:**
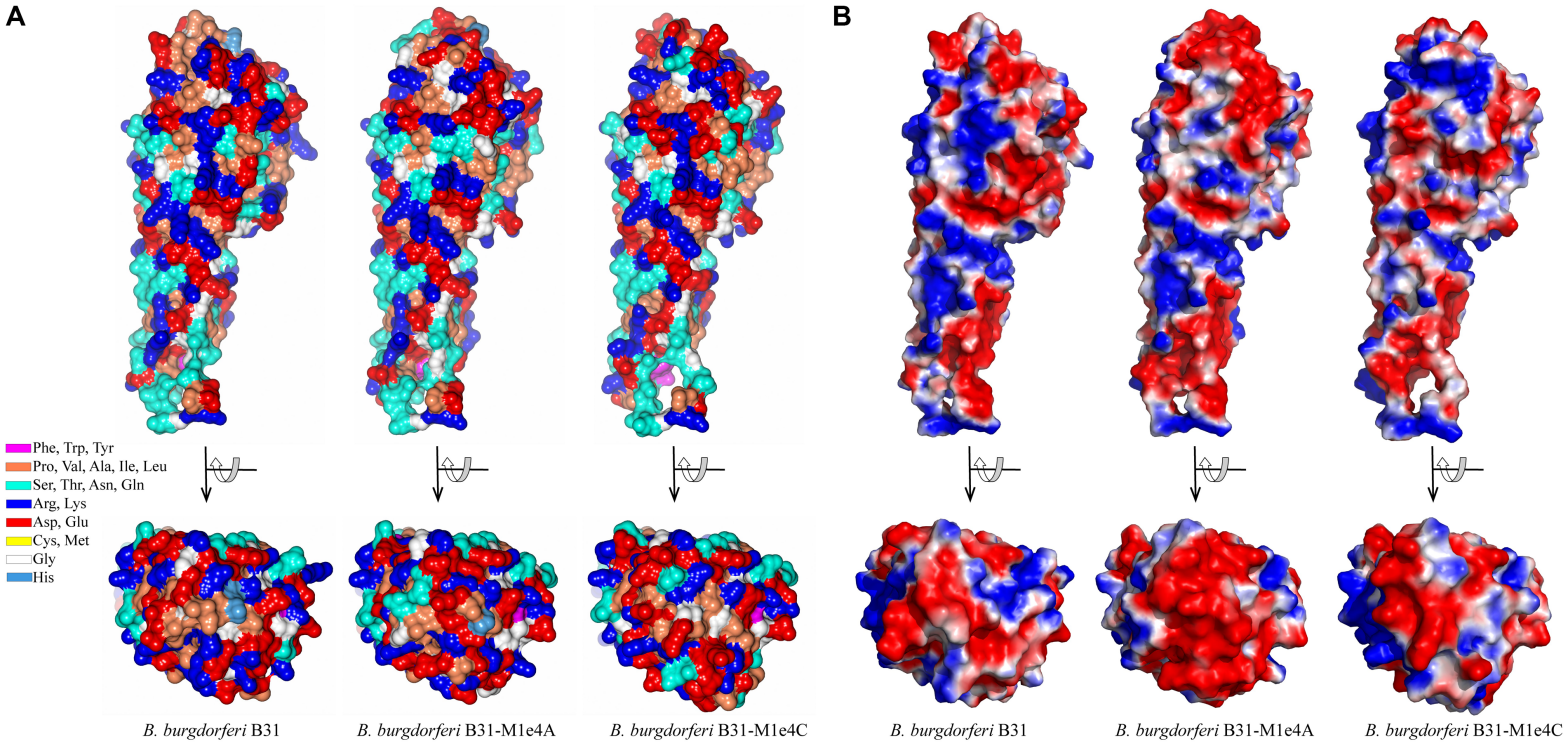
Comparison of the structures of three VlsE variants from *B. burgdorferi* B31, showing the retention of overall structure despite extensive variation in the VRs on the membrane distal surface. VlsE1 was the variant in the B31 parental strain used in mouse inoculation studies; M1e4A and M1e4C were VlsE sequences obtained from two clones isolated from the ear of a single mouse 4 weeks after infection with the VlsE1-expressing strain (Zhang et al., 1997; Zhang and Norris, 1998b). **(A)** Residue type illustration by color and **(B)** electrostatic potential surface maps of the AlphaFold predictions for VlsE1, M1e4a and M1e4c are shown as side and top (membrane distal surface) views. In **(B)**, red and blue coloration represent relative negative and positive surface potentials, respectively. The first 28 N-terminal residues (including the signal sequence, which was not part of the crystalized protein construct) were not resolved in the VlsE1 crystal structure and are excluded in this comparison.

We also examined the predicted effects of VR sequence variation within the constant background of the *B. burgdorferi* B31 VlsE protein by comparing the AlphaFold predicted structures of VlsE1 and two VlsE variants isolated following infection of mice with the VlsE1-expressing parental strain (Figure 10). The two isolates, M1e4A and M1e4C, were two clones obtained from the same ear biopsy of a C3HeN mouse infected by intradermal inoculation 4 weeks previously (Zhang et al., 1997; Zhang and Norris, 1998b). In optimized alignments, M1e4A and M1e4C differ from VlsE1 at 31 and 34 amino acid positions within the cassette region; M1e4A and M1e4C differ from each other at 27 positions. All but one of these AA differences (in M1e4A) are within the 6 variable regions. Despite these antigenic variation-derived differences, which result in both the apparent diversification of outward facing residues (Figure 10A) and the electrostatic surface potential differences (Figure 10B), the three B31 variants retain very similar overall structures. This high similarity is reflected by comparative RMSD values of 0.77 Å and 0.76 Å when VlsE1 was superimposed with M1e4A and M1e4C, respectively. Thus, the VRs can undergo extensive amino acid variation within the VRs on the membrane distal surface without affecting the global predicted structure of VlsE.

## 4 Discussion

### 4.1 Common properties of *vls* systems

The *vls* systems of LD *Borrelia* have a remarkable preservation of overall properties, but also possess differences that apparently do not interfere with the occurrence of sequence variation at the *vlsE* locus (Table 2). Taken together, this information provides valuable clues regarding the still mysterious gene conversion mechanism of this system. Some of the conserved properties appear to be unique to LD *Borrelia* systems; these include the head-to-head arrangement of *vlsE* and the *vls* silent cassette, the contiguous nature of the silent cassette array, the existence of a “mirrored” portion of the *vlsE* 5′ end on the cassette array, and the presence of inverted repeats in the region of the *vlsE*-silent cassette array junction. In some *vls* systems (those of *B. burgdorferi* strains B31 and PBae, *B. ba*var*iensis* PBaeII, and the two *B. mayonii* strains), there are two sets of inverted repeats within the intergenic region and in the mirrored *vlsE* 5′ ends (Supplementary Files 1, 2). It is likely that the inverted repeats lead to the formation of a cruciform structure that in some way facilitates the occurrence of gene conversion events (Chaconas et al., 2020). Indeed, in a *vls* mini system developed by Castellanos et al. (2018), the presence of the intergenic inverted repeat does increase *vlsE* recombination in the circular plasmid format. Prior studies have shown that *vlsE* located on a different plasmid from the one containing the *vls* silent cassette region does not undergo sequence variation during infection, and that *vlsE* sequence variation is necessary to promote survival of LD *Borrelia* in immunocompetent vertebrate hosts (Lawrenz et al., 2004; Bankhead and Chaconas, 2007; Rogovskyy et al., 2015). Other antigenic variation systems, such as those in RF *Borrelia*, *Neisseria* sp., *Anaplasma* sp., and *Trypanosoma* and *Plasmodium* organisms, have silent copies of antigenic variation protein genes or gene segments scattered in one or more plasmids or chromosomes (Centurion-Lara et al., 2004; Barbour et al., 2006; Dai et al., 2006; Vink et al., 2012; Palmer et al., 2016; Bangs, 2018; Lin et al., 2021). In some of these cases (e.g., RF *Borrelia* and *Trypanosoma*), a near complete copy of a silent antigenic variation protein gene can replace the one in the expression site, while in others (e.g., *Treponema pallidum*, *Neisseria* sp., *Anaplasma* sp., and *Plasmodium* sp.) the process resembles the random, segmental gene conversion that occurs during *vlsE* variation.

**Table 2:** Conserved characteristics of vls systems.

Properties maintained by purifying selection
Head-to-head arrangement of *vlsE* and *vlsS* cassette region
Inverted repeats at *vlsE*-*vls* cassette region junction
Preservation of sequence identical to 5′ end of *vlsE* at the beginning of the silent cassettes
Lack of promoter on 5′ *vlsE*-like sequence of silent cassettes
Contiguous array of *vlsS* silent cassettes
General preservation of the silent cassette region open reading frame and full-length cassettes
High G + C content and GC skew
Concentration of sequence heterogeneity within variable regions
Preservation of alpha helical structures despite the occurrence of constant region heterogeneity
Properties that are variable or not present
Lack of conservation of inverted repeat sequences or location
17-base pair direct repeats at either end of the cassettes are not present in most strains
There are no conserved arrangements of G-rich elements (such as G4 structures)
Intercassette frameshifts are common in some strains, absent in others
Intracassette stop codons and frameshifts are less common, but do occur
Truncations and internal deletions are present in a minority of silent cassettes

An important feature of the *vls* cassette regions is the high G + C content and GC skew, with the coding strand containing a high proportion of G residues. The average G + C value for the cassettes of the LD strains examined was 49.1% (Range 45–51.7%) (Table 1); in comparison, the G + C content (on the leading replication strand) of representative LD chromosomes is ~28.6% (Supplementary Figure S2), and of plasmids is 26–32%. Similarly, the average GC skew for *vlsS* cassettes was 0.52, whereas that of the LD chromosomal leading replication strand (which generally has a higher GC skew) was 0.18. Thus there must be strong selective pressure to maintain high G + C content and GC skew values in the *vls* cassette regions compared to the relatively low values that are found in the rest of the genomes of *Borrelia* organisms (including both LD and RF species and strains). It has been proposed that these unusual features are key to the *vlsE* gene conversion process (Norris, 2014; Chaconas et al., 2020). One possibility is that the high concentration of guanine residues on the coding strand facilitates the formation of G-quadruplex (G4) structures, which in turn may facilitate recombination between *vlsE* and silent cassette sequences. In its simplest form, four guanines from either the same (intramolecular) or different (intermolecular) polynucleotides form a planar G4 quartet through noncovalent Hoogsteen hydrogen bonds (Seifert, 2018). However, a stable G4 structure (G4-S) requires a minimum of three consecutive guanines on each strand, forming a box-like configuration; the strands containing these guanines can be either in a parallel or anti-parallel configuration (Seifert, 2018). Cahoon and Seifert (2009, 2013) have determined that the transcription of a small RNA near the *N. gonorrhoeae pilE* (pilin) gene facilitates the formation of a G4 structure, which in turn promotes the occurrence of *pilE* antigenic variation (Cahoon and Seifert, 2009; Cahoon and Seifert, 2013). This *N. gonorrhoeae* G4-S also binds the recombinase RecA, which is required for *pilE* gene conversion events (Kuryavyi et al., 2012). In a study regarding the potential involvement of G4-S in *vlsE* recombination, Walia and Chaconas (2013) focused on the 17 nt direct repeat region that flanks the *vls* cassette regions in *B. burgdorferi* B31, which contains a homopolymeric stretch of five guanines. They showed that this 17 nt sequence forms a complex with altered electrophoretic mobility *in vitro*, and that preservation of the consecutive run of 5 G’s is required for this activity. The occurrence of Hoogsteen G to G base pairing in this system was further substantiated by (1) the stability of this complex in the presence of K^+^ but not Li^+^ ions; and (2) the protection of the participating G residues from methylation by dimethyl sulfate. Walia and Chaconas (2013) further noted that clusters of 3–5 G’s are present at a very high frequency throughout the coding strand of the *vls* cassette sequences; the preponderance of G-rich codons contrasts with the AT-rich codons favored in the rest of the genome, indicative of strong positive selection of this characteristic in the *vls* cassette regions. The *vls* regions lack the compact arrangement of G clusters that could potentially form the stable, four-sided parallel strand “box” of the canonical G4 structure (as exemplified by the *N. gonorrhoeae* G4 sequence 5′-GGGTGGGTTGGGTGGG-3′) (Cahoon and Seifert, 2009; Walia and Chaconas, 2013; Chaconas et al., 2020). Nevertheless, the consistently high G content on the coding strand of the *vls* cassette sequences may give rise to noncanonical DNA structures that promote and stabilize strand invasion and thereby facilitate the *vlsE* gene conversion process. The underlying mechanisms of this facilitation remain to be determined.

Comparison of the *vlsS* cassettes in LD *Borrelia* strains also indicates the positive selection of diversified VR sequences, which would in turn increase the number of sequences available for VlsE protein sequence variation and hence immune evasion. In a prior study, Graves et al. (2013) examined the nonsynonymous/synonymous (dN/dS) ratio of codon differences occurring within the *vlsS* cassettes of 12 LD *Borrelia* strains. Within each of the 12 strains, the dN/dS ratio of codons in the six variable regions exceeded that of the surrounding constant regions (CRs), with the difference being statistically significant (*p* ≤ 0.05) in 10 of the 12 strains. Similarly, relative indels identified in the alignments of the silent cassettes within each strain were common in the variable regions, but rare in the constant regions (Graves et al., 2013). The indels are consistently in multiples of three bps, thus preserving the protein open reading frame. Graves et al. (2013) also found that indels were most common in areas where there were tandem repeat codons (e.g., a high frequency of GCT codons, encoding alanine). While these aspects were not examined in detail in the current study, our results are consistent with these prior findings.

The properties that vary among different strains are also informative with regard to the requirements for the occurrence of *vlsE* sequence variation (Table 2). For example, although inverted repeat sequences are consistently present at the *vlsE-vlsS* cassette region junction, there is little consistency in terms of the length, location, or sequences of the inverted repeats. The head-to-head arrangement with the consistent presence of the 5′ end sequence of *vlsE* on either side of the intervening sequence constitutes a portion of the inverted repeat sequence. However, some LD strains have multiple, nested inverted repeat regions, and as an extreme the two *B. mayonii* sequences (MN14-1420 and MN14-1539) have a central, perfect 122 bp palindrome in the middle of this region (Figure 6 and Supplementary File 1). Thus, the presence of inverted repeat sequences in this region appears to be important, but their exact nature and location is apparently not critical. Also, intercassette frameshifts are present in many strains but absent in others [e.g., see strains *B. garinii* Far04 (Figure 3B)] and *B. burgdorferi* 29805 (Supplementary File 2). Frameshifts within cassettes and stop codons are less common, but still occur. Clones containing the stop codon from *vlsS11* in *B. burgdorferi* B31 (Figure 3) have been isolated from infected mice (Zhang and Norris, 1998b). These results indicate that such mutated silent cassette regions can recombine into the *vlsE* expression site, and that organisms expressing “mutant,” truncated VlsE products can survive during infection (for at least a short period). Finally, silent cassettes that are truncated or have internal deletions are commonly present in *vlsS* arrays. The existence of strains containing the anomalies listed in Table 2 indicates that features such as a contiguous silent cassette region open reading frame are not required for *vlsE* sequence variation and survival of LD organisms.

### 4.2 Mechanisms of *vls* system diversification

As a prelude to this section, it is important to establish the difference between antigenic variation and heterogeneity. In the *vls* system, antigenic variation is a process in which portions of the *vlsE* cassette region are replaced with silent cassette segments of seemingly random length and location. This gene conversion process occurs within a given LD organism through a specialized mechanism in which most (but not all) of the nucleotide changes are templated from the silent cassettes (Coutte et al., 2009; Verhey et al., 2018b, 2019). Heterogeneity, however, refers to genetic differences between LD organisms that result from more general mechanisms, including mutational events, recombination, insertions/deletions, rearrangements, and horizontal DNA transfer mechanisms. This evolutionary diversification process occurs over longer time frames (e.g., hundreds of generations) and can affect all regions of *vlsE*, the intervening noncoding DNA region, the *vlsS* silent cassettes, and (hypothetically) any proteins or nucleotide elements involved in *vlsE* gene conversion.

As mentioned previously, the *vls* systems of most LD species and strains have diversified to the extent that there are rarely any clear evolutionary “pathways” that can be discerned. For example, the *vlsS* cassettes typically display no synteny, i.e., *vlsS1* of strain A is no more closely related to *vlsS1* of strain B than it is to any other of the *vlsS* cassettes of strain B. However, in this study we were able to identify several sets of related organisms that possessed sufficient sequence identity to provide clues regarding the evolutionary divergence of *vls* systems. These examples included: the occurrence of relative point mutations and short indels in the silent cassette regions of the closely related *B. burgdorferi* strains B31 and PAbe (Figure 4); the presence of internal duplication events in the silent cassette regions of *B. afzelii* Far04 (Figure 5) and *B. burgdorferi* 64b (Supplementary Figure S3); and the more complex duplication events and rearrangements discernable in the *vls* systems of the *B. mayonii* strains MN14-1539 and MN14-1420 (Figure 6). These findings certainly reinforce the description of LD *Borrelia* genetic material as “genomes in flux” by Casjens et al. (2000). They also suggest that there is strong positive selection for the divergence of many aspects of the *vls* locus, in line with the “balancing act” between diversification and maintenance of the properties required for effective *vlsE* gene conversion (Zhou and Brisson, 2014). All intracellular genetic change mechanisms appear to be active in *vls* system divergence, but we have been unable to identify any examples of apparent horizontal genetic transfer. The silent cassette array appears to be capable of considerable “accordion-like” expansion and contraction, with the number of silent cassettes varying between 13 and 24 in those sequences that include the complete locus (Supplementary Files 1, 2). Upper and lower limits in this variation are likely governed by having a sufficient pool of donor sequences for effective immune evasion vs. the genetic challenges to maintain a large, contiguous arrangement of related sequences.

### 4.3 *B. maritima* and *B. turcica—*examples of emerging (or senescent?) *vls*-like systems

Our analysis included two interesting cases that appear to represent antigenic variation systems that are either developing or decaying. *B. maritima* CA690 was isolated from an *Ixodes spinipalpis* tick in an estuarine region of Northern California (Margos et al., 2020b). The most common host for *I. spinipalpis* is the wood rat, and it is currently unknown if *B. maritima* infects humans. The *B. maritima vls* locus contains sequences that are closely related to those of other LD *Borrelia*. However, its arrangement is unique, having two head-to-head copies of partial *vlsE* sequences lacking part of the C-terminal encoding region (Figure 7). These are followed by a total of 10 *vls* sequence fragments, some of which are separated by frameshifts. No other *vls* sequences were found in the genome. The intervening region between the *vlsE-*like sequences lacks recognizable promoter or ribosome binding site sequences, so further studies would be needed to determine whether this locus expresses a protein. It is unclear at this point if this locus represents an emerging or decaying *vls* system.

*B. turcica* IST7 provides a very clearcut example of a novel, *vls-*like antigenic variation system in a relapsing fever group spirochete. This unique organism was isolated from a *Hyalomma aegyptium* tick on a tortoise in Turkey; it is part of a growing group of RF *Borrelia* associated with reptiles. The arrangement of the IST7 locus closely resembles the *vls* systems of LD *Borrelia*, with a head-to-head arrangement between the expression site and the array of silent cassettes (Figure 8). However, the predicted protein sequence of the expression site (*B. turcica* VlpE) more closely resembles the Vlps of RF *Borrelia*, including *B. coriaceae*, *B. tachyglossi*, and *B. miyamotoi* (Supplementary Figure S5). The *B. turcica* locus lacks the inverted repeats and *vlsE-*like 5′ end at the beginning of the silent cassette array that are characteristic of the LD *Borrelia vls* systems; also, 5′ and 3′ ends of the silent cassettes are not as consistent as found in most LD *vls* arrays. Finally, the *B. turcica vlp* sequences lack the prominent GC skew found consistently in *vls* systems, although they do have a relatively high G + C content. Taken together, this information indicates that it is unlikely that the *B. turcica vlp* system either evolved from a *vls* array present in an ancient common ancestor, or was acquired by horizontal transfer of a *vls* system from a Lyme disease spirochete. Rather, we propose that the *B. turcica* IST7 *vlp* system represents a remarkable example of convergent evolution. It will be of interest to see if other strains of this organism, or perhaps other organisms of the relapsing fever group, possess a similar system.

### 4.4 Preservation of VlsE structure despite framework heterogeneity and VR variation

We investigated the extent to which the AlphaFold predicted structure of VlsE was affected by the framework heterogeneity between species and strains (Supplementary Figure S1), and by the VR variation occurring within a given strain. VlsE structure was found to be remarkably similar regardless of differences introduced by either of these mechanisms (Figures 9; 10, Supplementary Figure S5). The preservation of the lipidated N-terminus that serves as the outer membrane anchor, the cluster of parallel *α*-helices that form the central portion of the protein, and the variability of the amino acids localized on the membrane distal surface indicates the importance of these attributes in VlsE function. These general attributes are also present in LD protein OspC (Kumaran et al., 2001), the RF protein Vsp1 (Lawson et al., 2006), and the predicted structures of RF Vlps; in contrast, the LD surface lipoproteins OspA and OspB (Becker et al., 2005; Makabe et al., 2006) that are predominant during the tick phase of infection and down-regulated during mammalian infection have primarily *β*-pleated sheet structures. This pattern implies that the general architecture of VlsE and related proteins is favorable in terms of interactions with antibodies and other host factors in the vertebrate environment.

There is agreement that a primary function of VlsE is immune evasion, through both its own antigenic variation and the at least partial shielding of other borrelial surface proteins (Xu et al., 2008; Rogovskyy and Bankhead, 2013; Norris, 2014; Rogovskyy et al., 2015; Bankhead, 2016; Batool et al., 2018; Rogovskyy et al., 2019; Chaconas et al., 2020; Lone and Bankhead, 2020). Studies regarding the antigenicity of VlsE variants indicate that the epitopes are not only dependent upon the localized effects resulting from 1 to 2 amino acid changes, but rather on alterations of the 3D conformation of VRs within the CR framework (Zhou and Brisson, 2017; Li et al., 2022).

Recent analyses have indicated that certain regions of VlsE are involved in self-dimerization and in dermatan sulfate binding. Verhey et al. (2019) implicated a noncontiguous set of 5 amino acids in dimerization, based in part on (a) their location at the interface between two VlsE monomers in the asymmetric unit of the crystal structure (Eicken et al., 2002) and (b) their nonvariance during antigenic variation in the strain *B. burgdorferi* JD1. However, the alignment of VlsE proteins from different organisms (Supplementary Figure S1) indicates that considerable heterogeneity is present in at least one of these amino acids (corresponding to E231 in JD1). In another study, VlsE was shown to be able to bind to the mammalian extracellular matrix (ECM) component dermatan sulfate (DS) (Tan et al., 2022). A group of four lysine residues on the α4 helix of VlsE of *B. burgdorferi* B31 was investigated for their role in DS binding, since positively charged amino acids such as lysine mediate this activity in other bacterial adhesins. Replacing all four lysines with methionine (resulting in a strain called B31-A VlsE_-ECM_) resulted in a greatly reduced ability of the strain to transiently bind to endothelial cells during mouse infection (Tan et al., 2022). Of the four lysines, only one (K169 in B31 VlsE1) is conserved in all 19 VlsE sequences from the different strains we examined (Supplementary Figure S1). It is possible that mutation of the codon encoding this lysine alone could cause the observed phenotype. Overall, it is important that relative homogeneity among strains as well as intrastrain variability be taken into consideration when investigating VlsE amino acids or regions potentially involved in functional activities.

### 4.5 The annotation issue

Since the first complete bacterial genome sequence became available in Fleischmann et al. (1995), the advent of next generation sequencing (NGS) approaches has resulted in a tremendous expansion in the number of available genome sequences. This wealth of information has greatly accelerated advances in our understanding of the correlation between genetics and phenotypic characteristics such as pathogenesis. An example of such an expansion is the recent completion of new genomic sequences of 44 strains from 19 different *Borreliella* species (Akther et al., 2024), which will be of great value in future analyses of *vls* systems as well as the Lyme disease field in general. This explosive increase in new data has also led to challenges in terms of genome sequence annotation. In most cases, the annotation of genes and other features has been handled by automated programs such as the NCBI Prokaryotic Genome Annotation Pipeline (PGAP) (Li et al., 2021; Haft et al., 2024). This automated platform has permitted the efficient annotation of protein and RNA product-encoding genes of ~36,000 new genomes per year. The resulting Reference Sequence (RefSeq) collection now also includes the protein family model (PFM) system that aids in the identification of functional groups. However, a shortcoming of the PGAP is that it recognizes only complete open reading frames; as a result, gene segments such as the *vls* silent cassettes are either poorly annotated (Figure 2) or not annotated at all. Some programs may be successful in identifying *vls* silent cassette regions (Li et al., 2022), but thus far automated systems are unable to identify features such as uneven or overlapping ends of, or frameshifts and stop codons within, silent cassette sequences. At least for now, the best system for delineating *vls* system structures is still the human mind, acknowledging that this procedure is a sometimes subjective and inexact science. Methods for making this process more systematic should be developed. In a more global sense, approaches for accurately annotating genetic elements (such as *vls* silent cassettes) that are important, but are not themselves genes, are needed.

## 5 Conclusion

In this study, we have examined the structural properties of *vls* antigenic variation systems and also predicted structure of 19 VlsE proteins. With this focus, we have not delved to any great extent into the considerable information available regarding the antigenicity of VlsE and enzymatic activities involved in the *vlsE* gene conversion process. We apologize if any references pertinent to this study were not cited.

The *vls* system exemplifies the evolution of a remarkably complex process that permits a pathogen to evade the immune system of its mammalian host. Diverse antigenic variation systems have evolved independently in a wide range of pathogenic organisms, including *Neisseria*, *Treponema*, *Anaplasma*, *Trypanosoma*, *Plasmodium* and many other bacterial and protozoal genera. All of these systems must have arisen from relatively simple genetic loci, e.g., through duplications of a surface protein gene with subsequent divergence of the copies. Antigenic variation is particularly common in organisms that cause persistent infections that can last weeks, months, or years. Positive selection is provided by the need for prolonged presence of the organism to permit transmission and hence survival, as in the case of Lyme disease *Borrelia* in their complex mammal-tick transmission cycle. This study confirms the positive selection of the common properties of *vls* loci (Table 2), which will hopefully provide information valuable in further delineating the *vlsE* gene conversion process and, perhaps, mechanisms for inhibiting immune evasion. The novel *B. maritima* and *B. turcica vls*-like systems indicate that nascent immune evasion mechanisms are continuing to develop, and help to provide insight into this evolutionary process.

## Data Availability

The original contributions presented in the study are included in the article/Supplementary material, further inquiries can be directed to the corresponding author.

## Footnotes

^1^www.ncbi.nlm.nih.gov

^2^https://itol.embl.de/

^3^http://www.bioinformatics.nl/emboss-explorer/

^4^http://tubic.tju.edu.cn/Ori-Finder/
