# Supplementary material for: Meta-analysis of the Vmp-like sequences of Lyme disease Borrelia: evidence for the evolution of an elaborate antigenic variation system
Source: Front Microbiol. 2024 Oct 10;15:1469411. doi: 10.3389/fmicb.2024.1469411 (PMC11499132; doi:10.3389/fmicb.2024.1469411)
Supplement: Supplementary file 2 [file Data_Sheet_2.PDF]

Supplemental file 2. Maps of the vls regions included in this study, in the order shown in Table 1. Silent cassettes are labeled in an abbreviated format (e.g. *S1* = *vlsS1*).

Key:

- ▲ **Frameshift within cassette (intracassette)**
- ▲ **Frameshift between cassettes (intercassette)**
- * **Stop codon within cassette (intracassette)**
- * **Stop codon between cassettes (intercassette)**
- ▬ **Non-cassette *vlsE* (or *vlsE*-like) sequences**
- ⟶ **Inverted repeats**

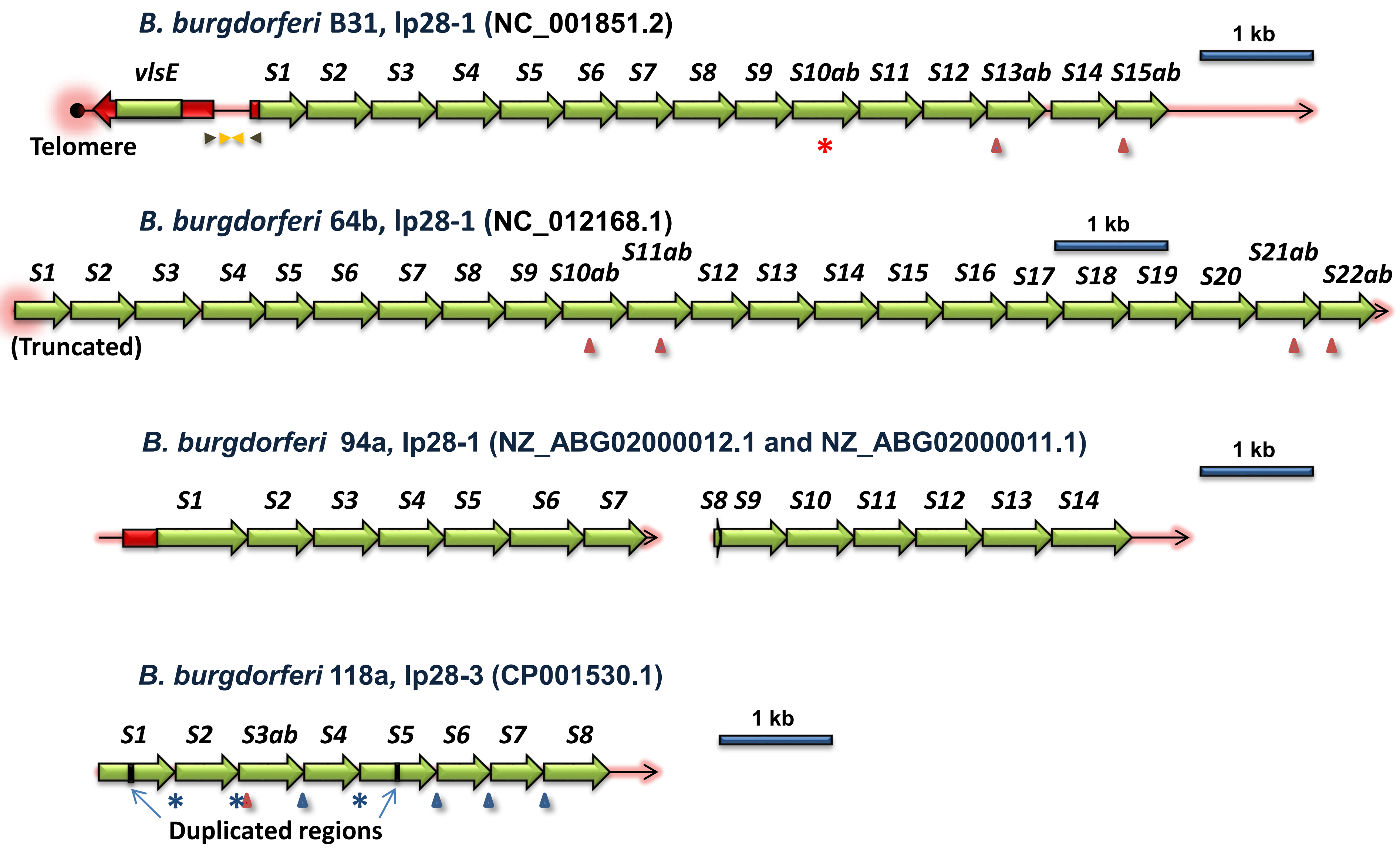
B. burgdorferi B31, lp28-1 (NC_001851.2)
1 kb
vlsE
S1 S2 S3 S4 S5 S6 S7 S8 S9 S10ab S11 S12 S13ab S14 S15ab
Telomere
B. burgdorferi 64b, lp28-1 (NC_012168.1)
1 kb
S1 S2 S3 S4 S5 S6 S7 S8 S9 S10ab S11ab S12 S13 S14 S15 S16 S17 S18 S19 S20 S21ab S22ab
(Truncated)
B. burgdorferi 94a, lp28-1 (NZ_ABG02000012.1 and NZ_ABG02000011.1)
1 kb
S1 S2 S3 S4 S5 S6 S7
S8 S9 S10 S11 S12 S13 S14
B. burgdorferi 118a, lp28-3 (CP001530.1)
1 kb
S1 S2 S3ab S4 S5 S6 S7 S8
Duplicated regions

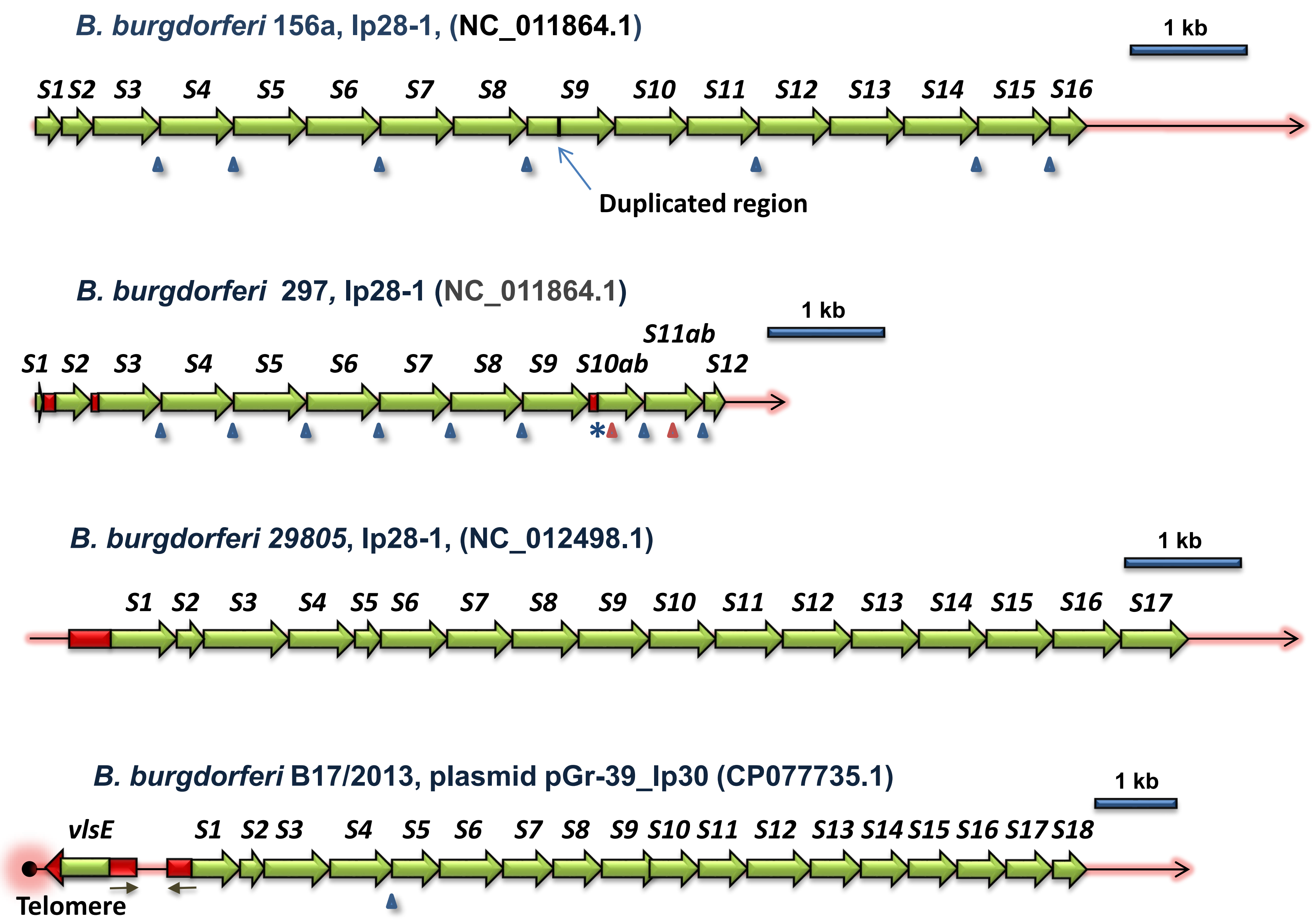
B. burgdorferi 156a, lp28-1, (NC_011864.1)
1 kb
S1 S2 S3 S4 S5 S6 S7 S8 S9 S10 S11 S12 S13 S14 S15 S16
Duplicated region
B. burgdorferi 297, lp28-1 (NC_011864.1)
1 kb
S11ab
S1 S2 S3 S4 S5 S6 S7 S8 S9 S10ab S12
*
B. burgdorferi 29805, lp28-1, (NC_012498.1)
1 kb
S1 S2 S3 S4 S5 S6 S7 S8 S9 S10 S11 S12 S13 S14 S15 S16 S17
B. burgdorferi B17/2013, plasmid pGr-39_lp30 (CP077735.1)
1 kb
vlsE
S1 S2 S3 S4 S5 S6 S7 S8 S9 S10 S11 S12 S13 S14 S15 S16 S17 S18
Telomere

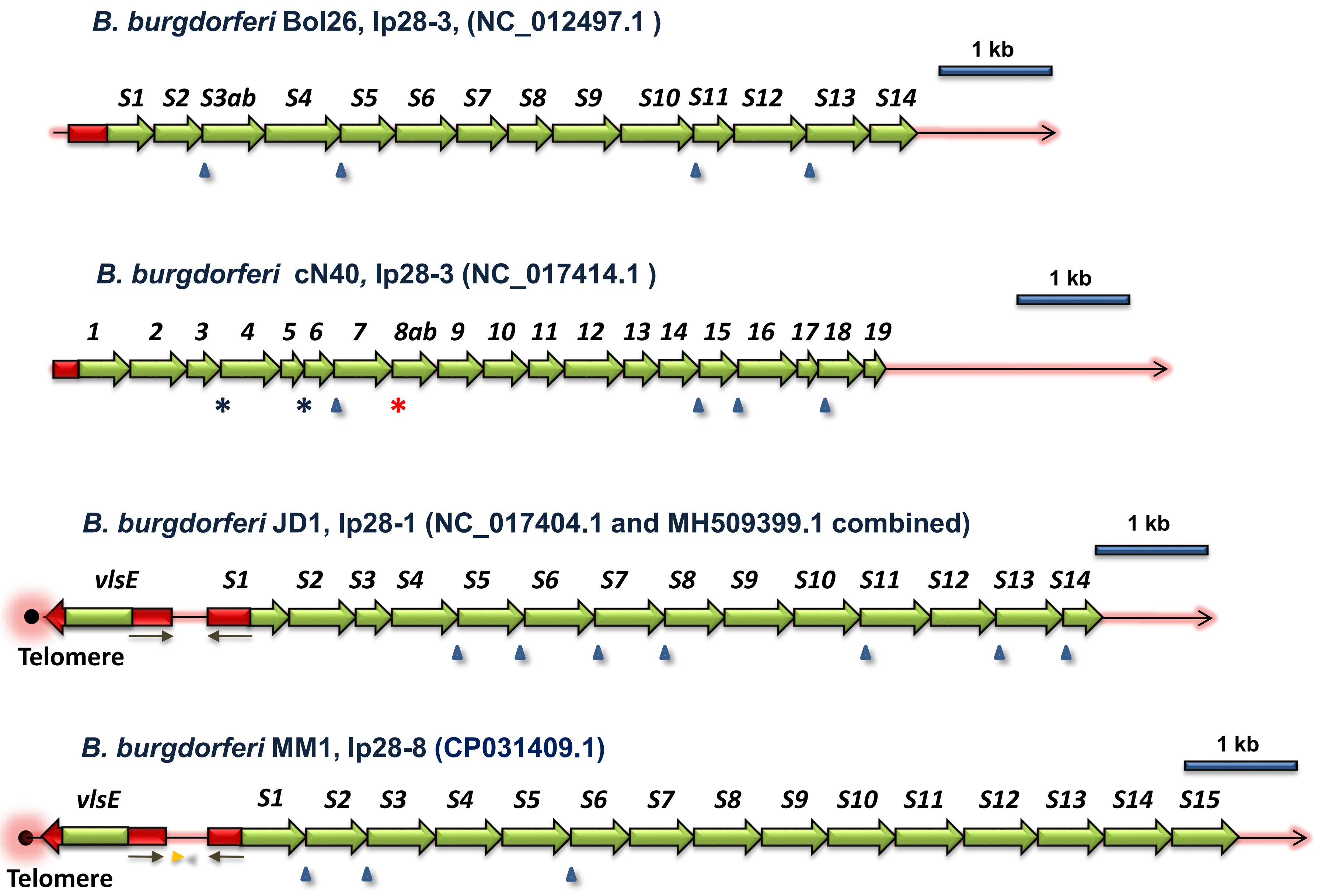
B. burgdorferi Bol26, lp28-3, (NC_012497.1)
1 kb
S1 S2 S3ab S4 S5 S6 S7 S8 S9 S10 S11 S12 S13 S14
B. burgdorferi cN40, lp28-3 (NC_017414.1)
1 kb
1 2 3 4 5 6 7 8ab 9 10 11 12 13 14 15 16 17 18 19
B. burgdorferi JD1, lp28-1 (NC_017404.1 and MH509399.1 combined)
1 kb
vlsE S1 S2 S3 S4 S5 S6 S7 S8 S9 S10 S11 S12 S13 S14
Telomere
B. burgdorferi MM1, lp28-8 (CP031409.1)
1 kb
vlsE S1 S2 S3 S4 S5 S6 S7 S8 S9 S10 S11 S12 S13 S14 S15
Telomere

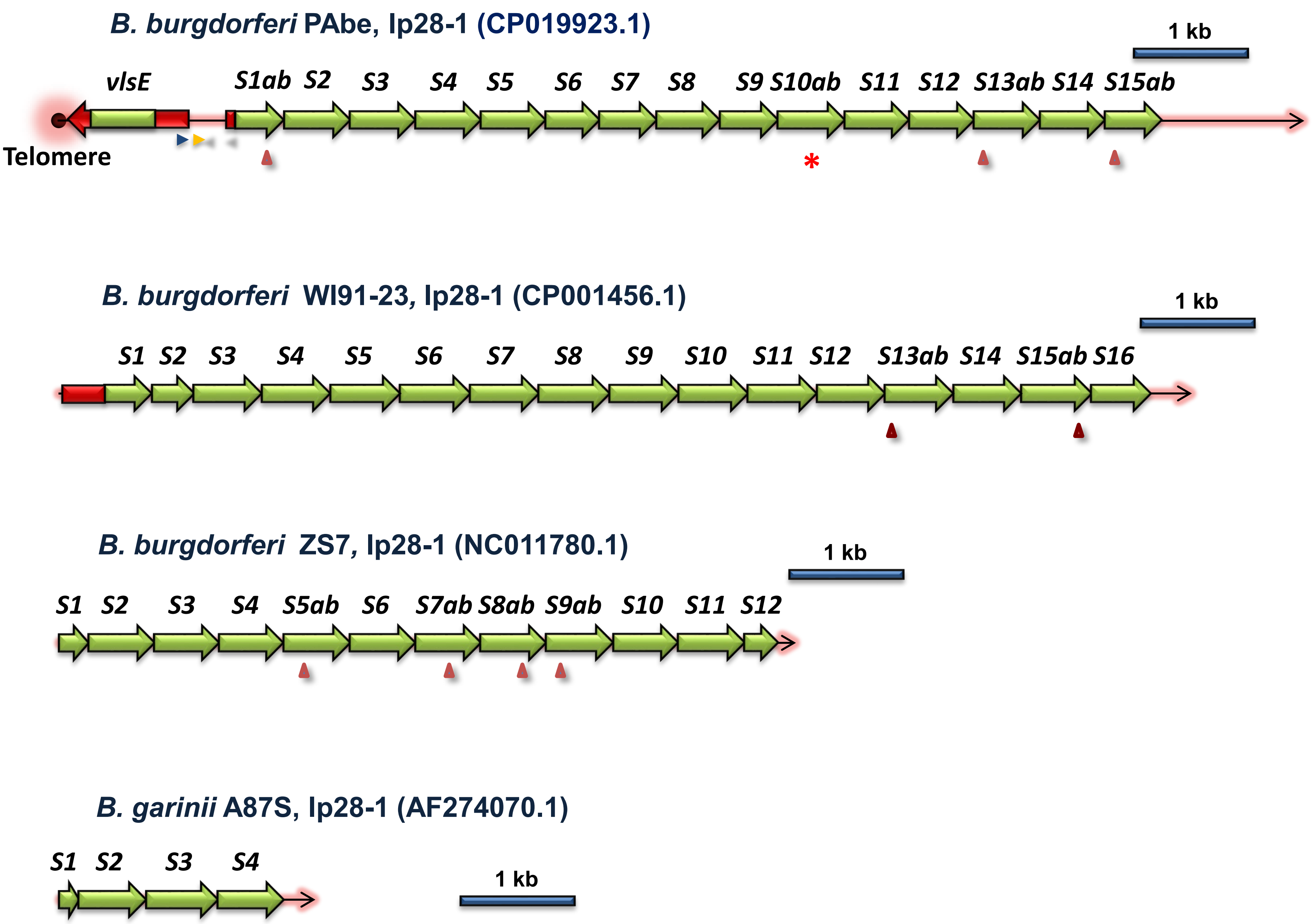

B. burgdorferi PAbe, lp28-1 (CP019923.1)
1 kb
vlsE
S1ab S2 S3 S4 S5 S6 S7 S8 S9 S10ab S11 S12 S13ab S14 S15ab
Telomere
*
B. burgdorferi WI91-23, lp28-1 (CP001456.1)
1 kb
S1 S2 S3 S4 S5 S6 S7 S8 S9 S10 S11 S12 S13ab S14 S15ab S16
B. burgdorferi ZS7, lp28-1 (NC011780.1)
1 kb
S1 S2 S3 S4 S5ab S6 S7ab S8ab S9ab S10 S11 S12
B. garinii A87S, lp28-1 (AF274070.1)
S1 S2 S3 S4
1 kb

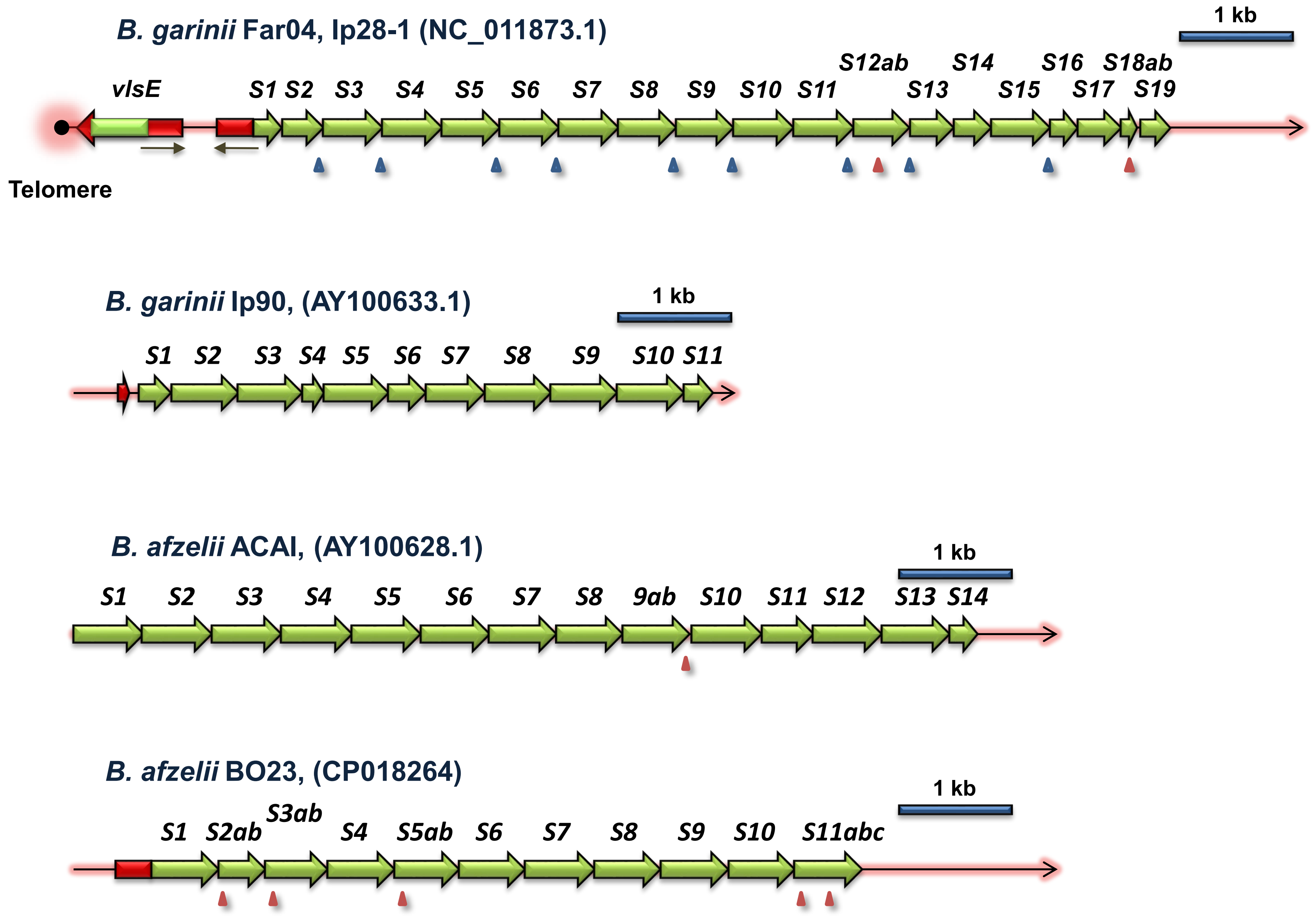
B. garinii Far04, lp28-1 (NC_011873.1)
1 kb
vlsE
S1 S2 S3 S4 S5 S6 S7 S8 S9 S10 S11 S12ab S13 S14 S15 S16 S17 S18ab S19
Telomere
B. garinii lp90, (AY100633.1)
1 kb
S1 S2 S3 S4 S5 S6 S7 S8 S9 S10 S11
B. afzelii ACAI, (AY100628.1)
1 kb
S1 S2 S3 S4 S5 S6 S7 S8 9ab S10 S11 S12 S13 S14
B. afzelii BO23, (CP018264)
1 kb
S1 S2ab S3ab S4 S5ab S6 S7 S8 S9 S10 S11abc

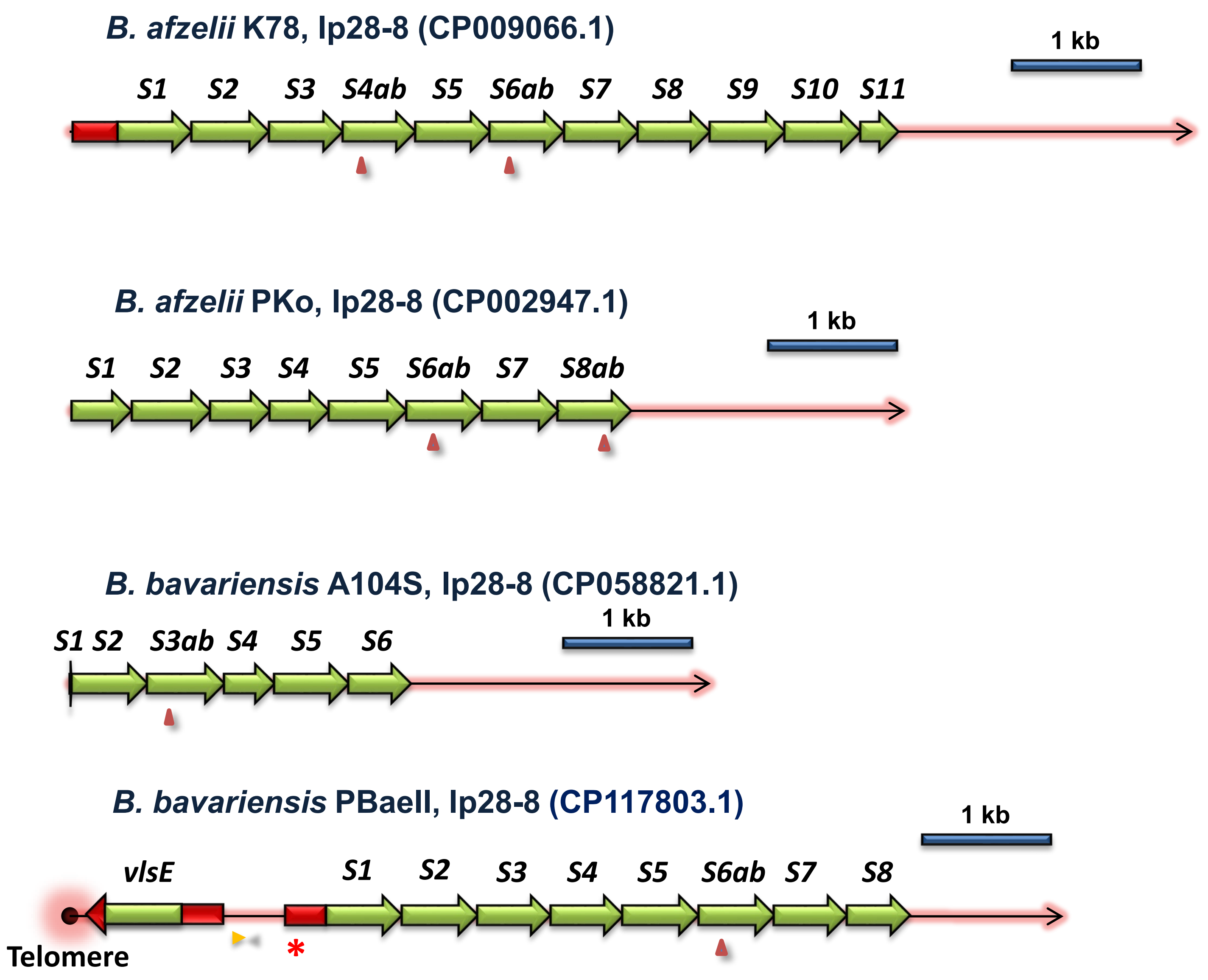
B. afzelii K78, lp28-8 (CP009066.1)
1 kb
S1 S2 S3 S4ab S5 S6ab S7 S8 S9 S10 S11
B. afzelii PKo, lp28-8 (CP002947.1)
1 kb
S1 S2 S3 S4 S5 S6ab S7 S8ab
B. bavariensis A104S, lp28-8 (CP058821.1)
1 kb
S1 S2 S3ab S4 S5 S6
B. bavariensis PBaeII, lp28-8 (CP117803.1)
1 kb
vlsE
S1 S2 S3 S4 S5 S6ab S7 S8
*
Telomere

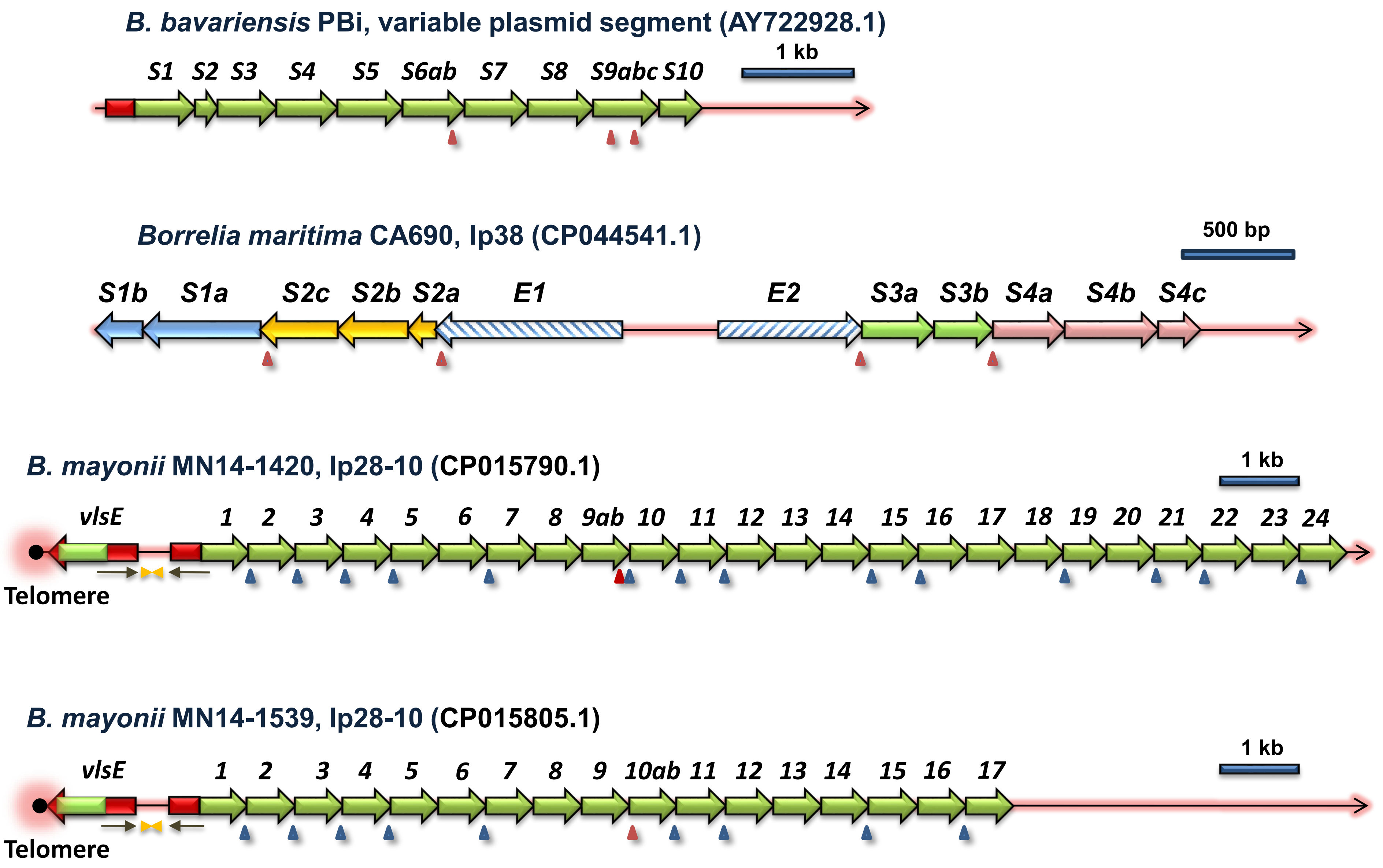
B. bavariensis PBi, variable plasmid segment (AY722928.1)
1 kb
S1 S2 S3 S4 S5 S6ab S7 S8 S9abc S10
Borrelia maritima CA690, lp38 (CP044541.1)
500 bp
S1b S1a S2c S2b S2a E1 E2 S3a S3b S4a S4b S4c
B. mayonii MN14-1420, lp28-10 (CP015790.1)
1 kb
vlsE
1 2 3 4 5 6 7 8 9ab 10 11 12 13 14 15 16 17 18 19 20 21 22 23 24
Telomere
B. mayonii MN14-1539, lp28-10 (CP015805.1)
1 kb
vlsE
1 2 3 4 5 6 7 8 9 10ab 11 12 13 14 15 16 17
Telomere

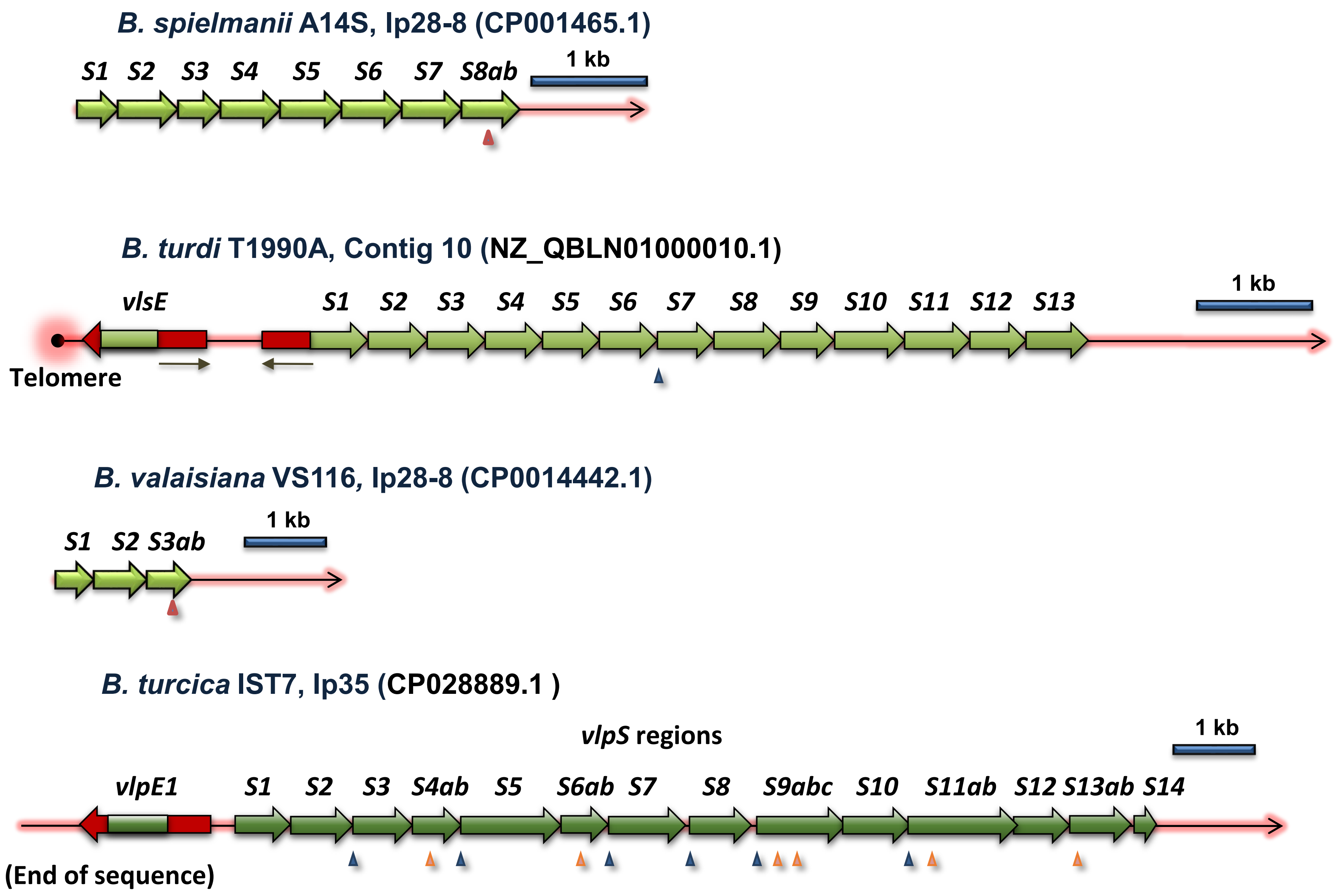

B. spielmanii A14S, lp28-8 (CP001465.1)
S1 S2 S3 S4 S5 S6 S7 S8ab
1 kb
B. turdi T1990A, Contig 10 (NZ_QBLN01000010.1)
vlsE
S1 S2 S3 S4 S5 S6 S7 S8 S9 S10 S11 S12 S13
1 kb
Telomere
B. valaisiana VS116, lp28-8 (CP0014442.1)
S1 S2 S3ab
1 kb
B. turcica IST7, lp35 (CP028889.1)
vlpS regions
1 kb
vlpE1
S1 S2 S3 S4ab S5 S6ab S7 S8 S9abc S10 S11ab S12 S13ab S14
(End of sequence)
