# Supplementary material for: Meta-analysis of the Vmp-like sequences of Lyme disease Borrelia: evidence for the evolution of an elaborate antigenic variation system
Source: Front Microbiol. 2024 Oct 10;15:1469411. doi: 10.3389/fmicb.2024.1469411 (PMC11499132; doi:10.3389/fmicb.2024.1469411)
Supplement: Supplementary file 3 [file Presentation_1.pdf]

Supplementary Materials

Supplemental File 1. Coordinates and properties of *vls* systems of LD *Borrelia* organisms and the *vls*-like system of the RF organism *Borrelia turcica*. (separate file)

Supplemental File 2. Maps of the *vls* regions included in this study, in the order shown in Supplemental File 1. (separate file)

Figure S1. Sequence alignment of the VlsE proteins from strains of *B. burgdorferi* (B_bu), *B. turdi* (B_tu), *B. afzelii* (B_af), *B. garinii* (B_ga), *B. mayonii* (B_ma), *B. finlandensis* (B_fi), and *B. spielmanii* (B_sp). The residues identical in all the VlsE proteins are colored with a red background, while residues with similar amino acids have red lettering. The variable regions (VR1 to VR6) are illustrated with light green (VR1), violet (VR2), yellow (VR3), blue (VR4), dark green (VR5), and dark purple (VR6) backgrounds. The light brown background shows a region of heterogeneity between the LD *Borrelia* strains that is distinct from the VRs. The C-terminal sequence is also a region of heterogeneity. The positions of α-helices and β-pleated sheets (based on the *B. burgdorferi* B31 VlsE1 crystal structure) are indicated.

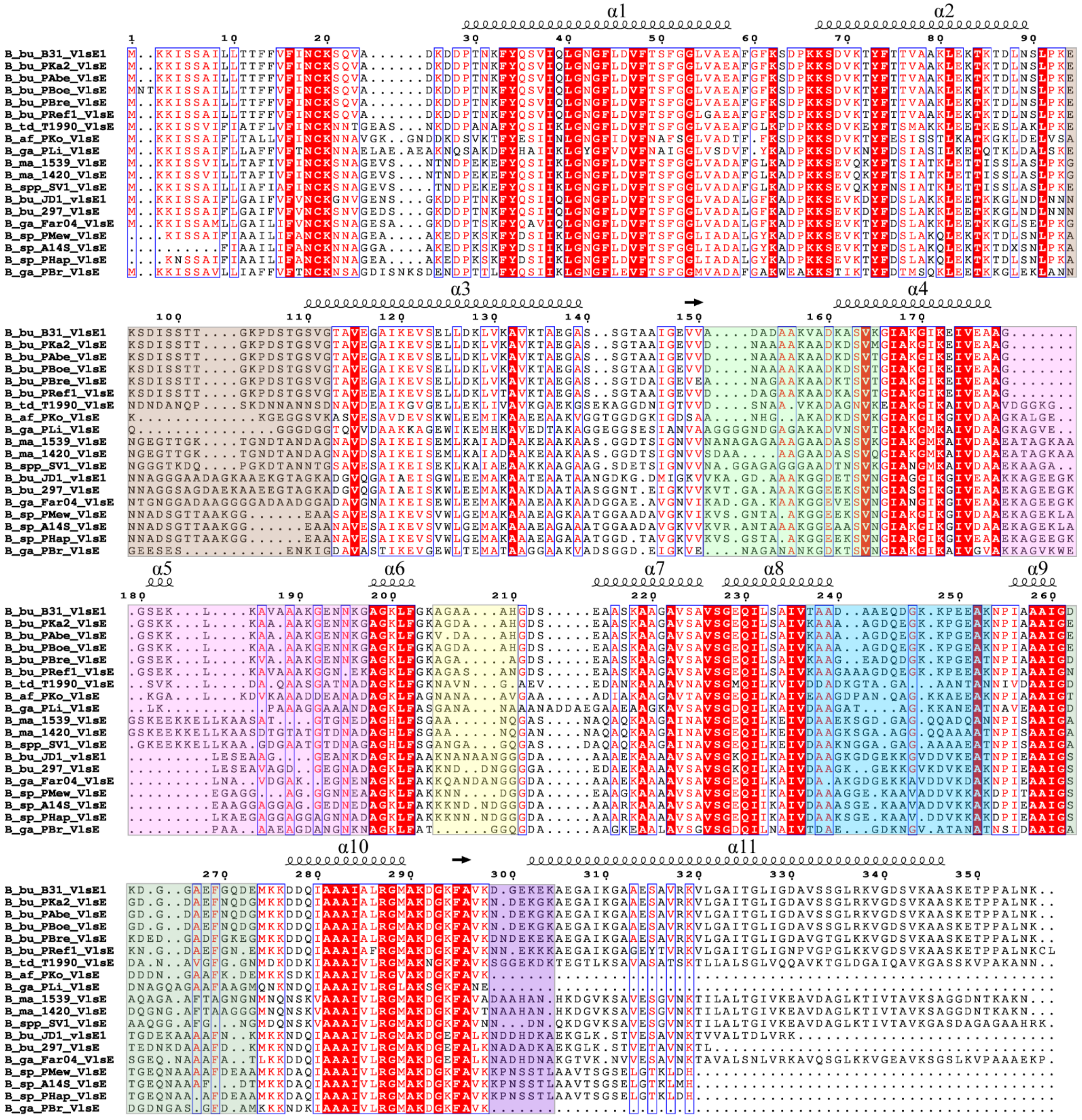

Figure S2. *vls* sequences have unusually high G+C content and GC skew. Selected *vlsS*, *vlsE*, *vlsH* (*vls* homolog), relapsing fever variable large protein (*vlp*) and variable small protein (*vsp*) sequences, and chromosomal values are shown. LB = Lyme Borreliosis; RF = Relapsing Fever. The *vlsS* G+C and GC skew values are higher than are the *vlsE* values because the elevation in these values is limited to the cassette region of *vlsE*.

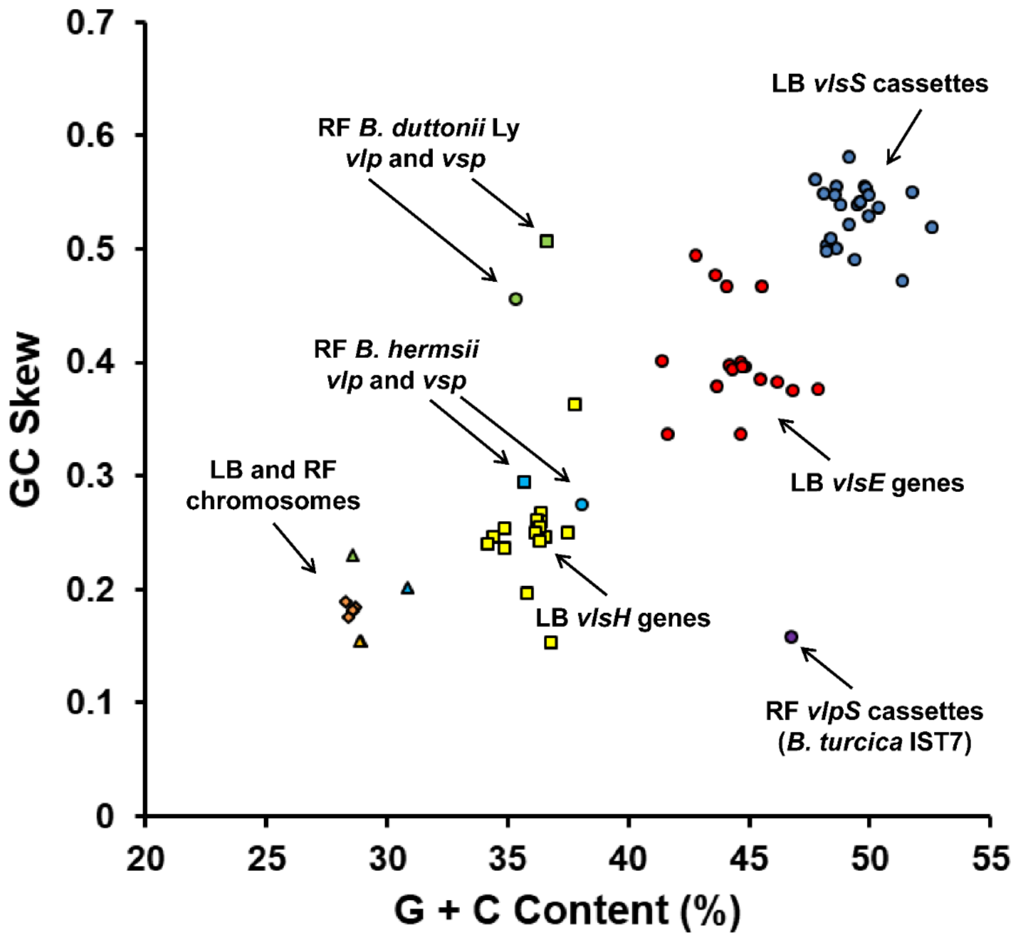

Figure S3. *vls* locus of *B. burgdorferi* strain 64b, showing the presence of regions of sequence identity (ROIs) resulting from duplication events. Note that the 64b locus sequence is incomplete, lacking *vlsE* and likely additional silent cassettes on the 5' end of what is currently termed *vlsS1*.

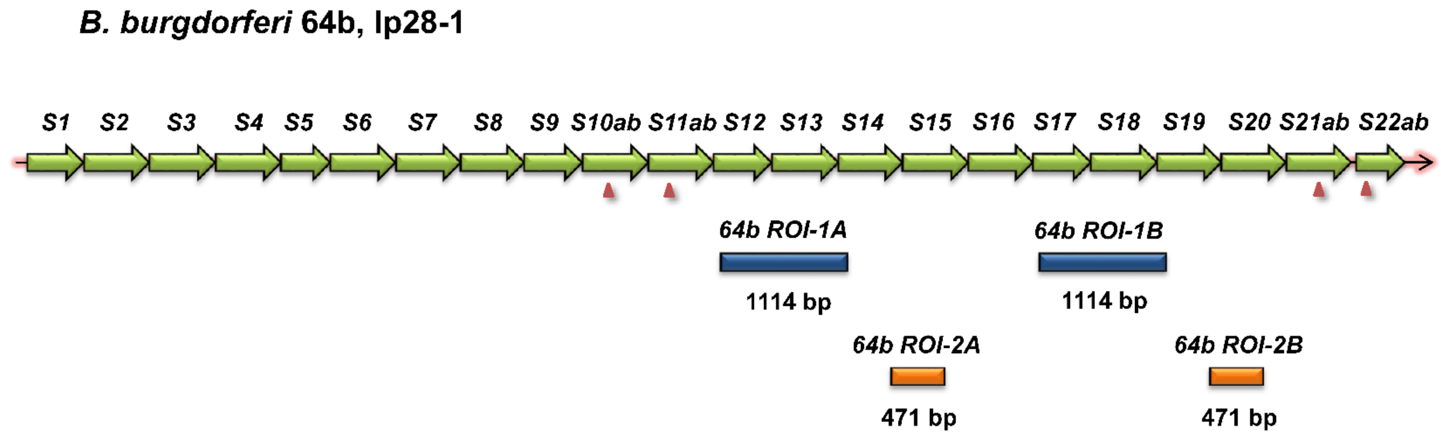

Figure S4. *vls* silent cassette nucleotide sequences of the *B. mayonii* strains MN14-1539 and MN-1420 exhibit a high degree of sequence identity, as shown in this unrooted phylogenetic tree presentation. Branch lengths are shown as numerical values; values of zero in the terminal branches indicate sequence identity. In some cases silent cassettes are identical both within a strain (consistent with duplication events) and between strains (indicating common ancestry); such an occurrence is exemplified by the cluster of identical silent cassettes 1539 *vlsS8,* 1539 *vlsS13*, 1420 *vlsS12* and 1420 *vlsS17* in the upper right corner of the diagram.

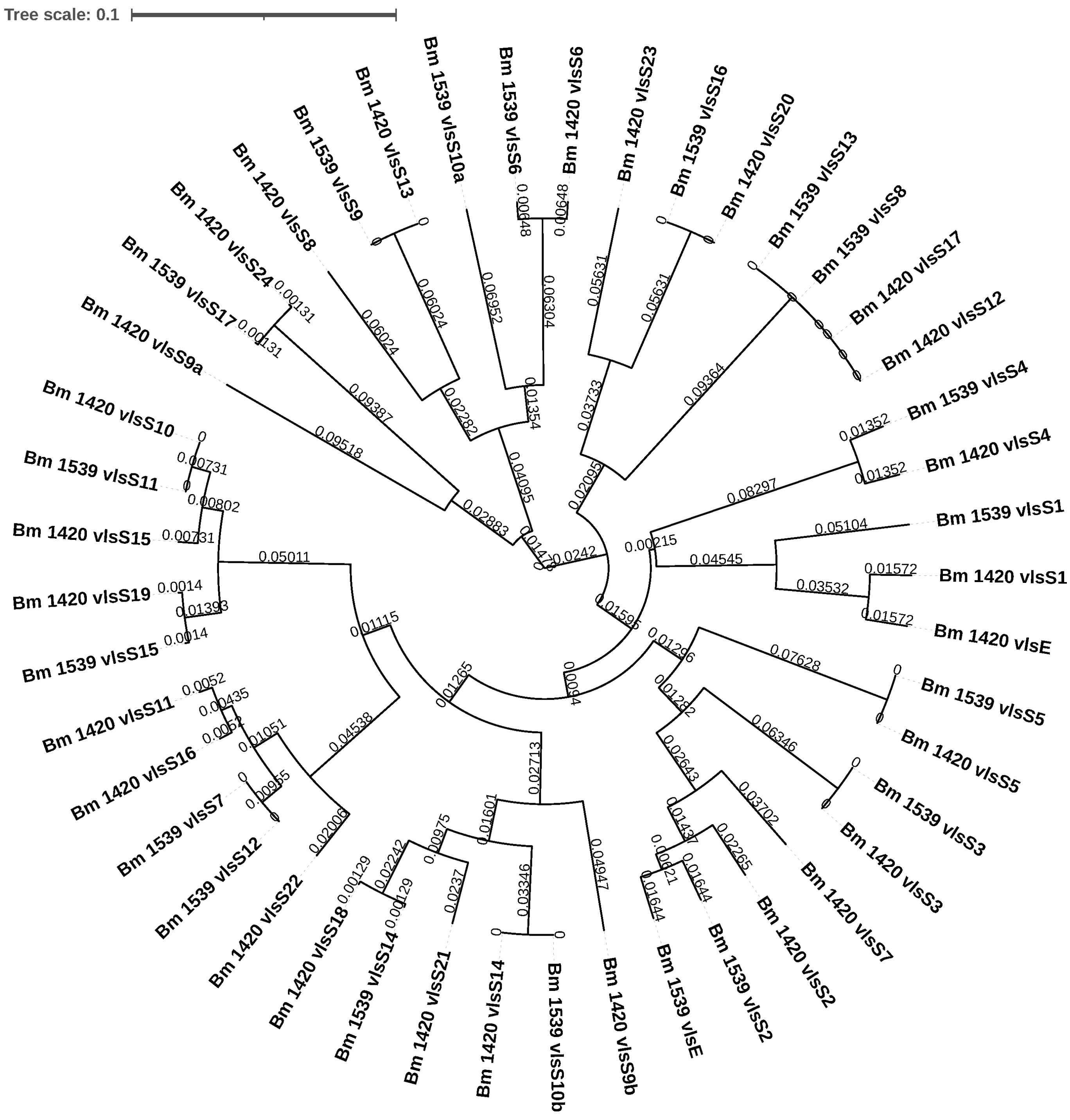

Figure S5. Varied degrees of relatedness among Lyme disease *Borrelia* VlsE and relapsing fever *Borrelia* Vlp predicted amino acid sequences as depicted in an unrooted phylogenetic tree format. Examples of closely related VlsE sequences include the cluster of "B31-like" *B. burgdorferi* strains in the lower left, the two *B. mayonii* strains at the lower right, and the three *B. spielmanii* strains on the right side. The relapsing fever *Borrelia* Vlp sequences (upper left) exhibit greater heterogeneity, and the VlpE1 sequence of *B. turcica* IST7 is included within this group. Species name abbreviations: af = *afzelii*, bu = *burgdorferi*, co = *coriaceae*, ga = *garinii*, he = *hermsii*, ma = *mayonii*, mi = *miyamotoi*, sp = *spielmanii*, ta = *tachyglossi*, td = *turdi*, tr = *turcica*, va = *valaisiana*.

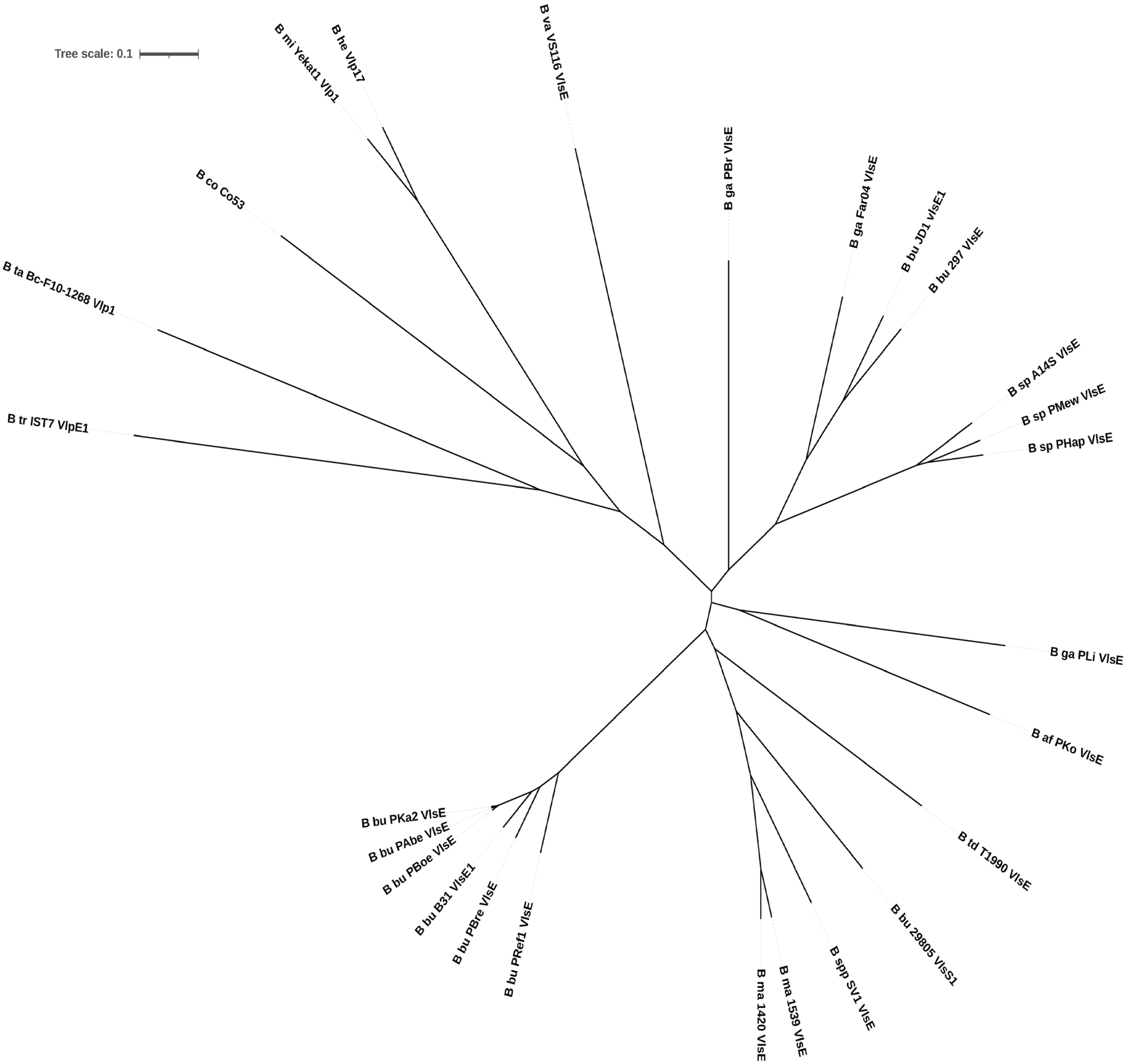

Figure S6. Variability of the electrostatic potential surface patterns of the VlsE AlphaFold-predicted 3D structures from 19 LD *Borrelia* strains. The variable regions (VRs) are depicted as a space-filling model showing the electrostatic potential, whereas the constant regions (CRs) are shown as a ribbon model. Red coloration corresponds to negatively charged areas, and blue coloration to positively charged regions.

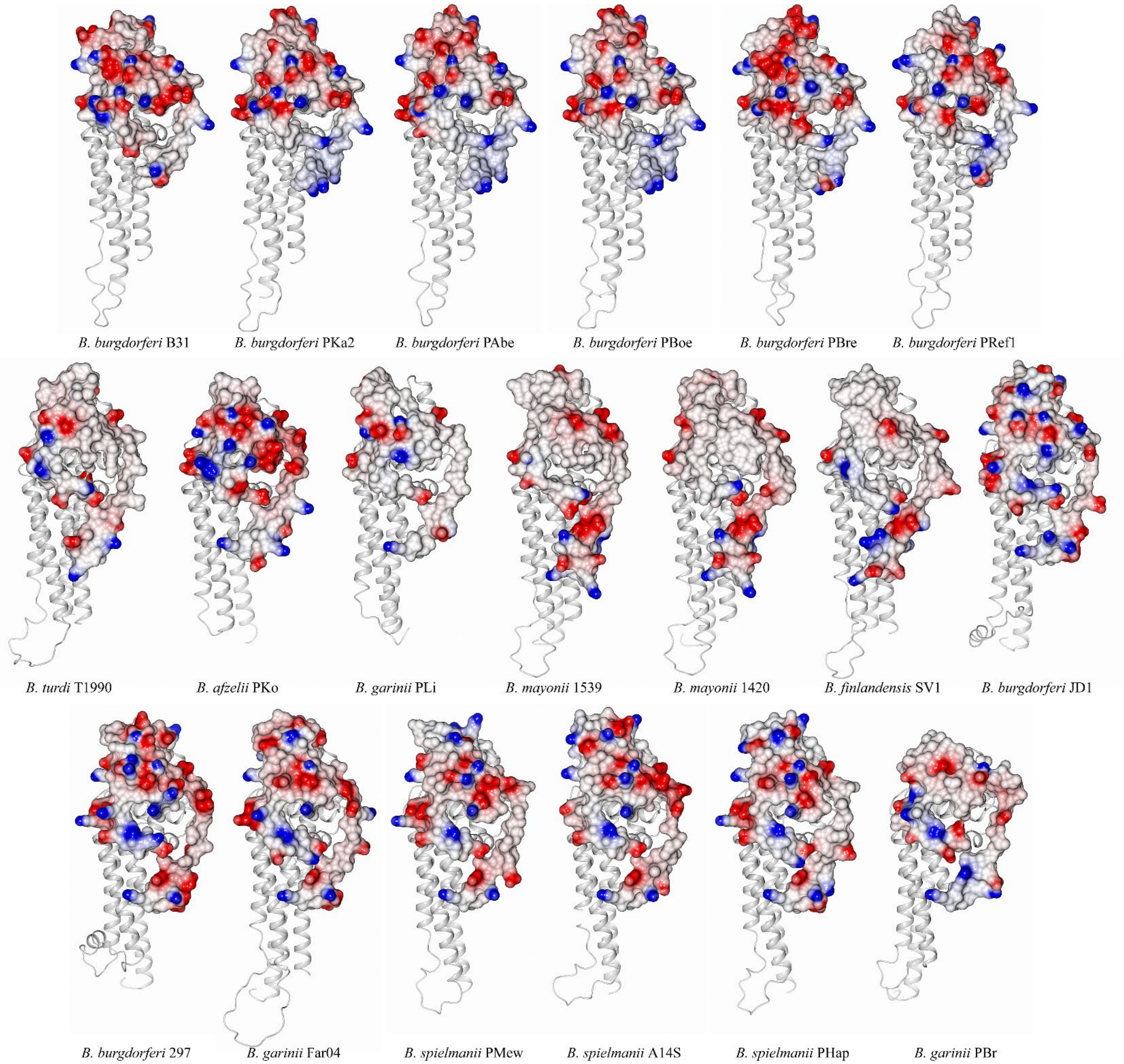

Table S1. Properties of *vlsE* genes examined in this study.

Species	Strain	GenBank DNA	Protein	Nt	AA	%GC	GC skew	AT skew	%AA Id/ Sim[a]	pI	Site of isolation	Specimen	Notes
B. burgdorferi	B31	U76405.1	AAC45733.1	1071	357	45	0.40	0.06	(100/100)	8.30	Shelter Island, NY, USA	*I. scapularis* tick	
B. burgdorferi	PAbe	AJ850088.1	CAH61548.1	1065	355	44	0.39	0.07	92/94	8.87	Germany	Human CSF	
B. burgdorferi	PBoe	AJ850089.1	CAH61549.1	1074	358	45	0.40	0.06	93/94	8.87	Germany	Human synovial fluid	
B. burgdorferi	PBre	AJ630107.1	CAF34025.1	1068	356	44	0.40	0.08	91/93	7.88	Germany	Human skin	
B. burgdorferi	PKa2	AJ630111.1	CAF34029.1	1068	356	45	0.40	0.06	93/95	8.87	Germany	Human CSF	
B. burgdorferi	PRef1	AJ630112.1	CAF34030.1	1077	359	44	0.38	0.07	88/90	8.99	Germany	Human CSF	
B. burgdorferi	297	AY052626.1	AAL13334.1	1017	339	43	0.49	0.09	46/53	5.03	Connecticut, USA	Human CSF	
B. burgdorferi	29805	NC_012498.1	*vlsS1*+5' end	906	302	42	0.34	0.01	43/51	7.08	Connecticut, USA	--	Partial
B. afzelii	PKo	AJ630109.1	CAF34027.1	894	298	44	0.48	0.01	41/47	7.07	Germany	Human skin	Partial
B. garinii	Far04	NC_011873.1	WP_012622732.1	1125	375	45	0.47	0.04	49/59	5.41	Faroe Islands, Denmark	Puffin blood	
B. garinii	PBr	AJ630108.1	CAF34026.1	897	299	41	0.40	0.07	38/42	5.26	Germany	Human CSF	Partial
B. garinii	PLi	AJ630110.1	CAF34028.1	909	303	45	0.38	0.00	35/42	5.18	Germany	Human CSF	Partial
B. spielmanii	A14s	AM993151.1	CAQ52813.1	978	326	48	0.38	0.12	40/49	8.34	Netherlands	Human skin	Partial
B. spielmanii	PHap	AM993152.1	CAQ52814.1	996	332	46	0.38	0.14	35/41	8.77	Germany	Human skin	Partial
B. spielmanii	PMew	AM993153.1	CAQ52815.1	984	328	47	0.38	0.12	41/49	8.33	Germany	Human skin	Partial
B. valaisiana	VS116	CP001440.1	ACN52946.1	1185	395	37	0.25	0.17	33/41	7.89	Switzerland	*I. ricinus* tick	
B. finlandensis	SV1	NZ_ABJZ02000007.1	WP_008882912.1	1116	372	45	0.34	0.05	46/57	7.96	Finland	*I. ricinus* tick	

a Percent amino acid identity/similarity with B31 VlsE1

Table S2. *vls* homologs (*vlsH*) of Lyme disease *Borrelia* examined in this study. Full-length *vlsH* genes encode a 369-375 amino acid lipoprotein that has sequence similarity to VlsE. Numbers (e.g. *vlsH1*, *vlsH2*) indicate the existence of different *vlsH* homologs in that strain. Letters (such as *vlsH1a*, *vlsH1b*) refer to fragments of the gene separated by frameshifts. Segments resulting from common frameshifts are highlighted.

Species	Strain	Replicon	Description	Gene	Protein	DNA	Orientation	5'	3'	Length[a]	AA
B. burgdorferi	156a	lp28-6	vlsH1	Bb156a_Z01	WP_012622681.1	NC_011872.1	Reverse	117	1226	1110	370
B. burgdorferi	29805	lp38	vlsH1a	--	--	CP001548.1	Reverse	38222	38389	168	56
B. burgdorferi	29805	lp38	vlsH1b	--	--	CP001548.1	Reverse	37824	38222	399	133
B. burgdorferi	29805	lp38	vlsH1c	--	--	CP001548.1	Reverse	37661	37825	165	55
B. burgdorferi	29805	lp38	vlsH1d	--	--	CP001548.1	Reverse	37285	37662	378	126
B. burgdorferi	64b	lp28-4	vlsH1	BBU64B_I0038	WP_012665272.1	NC_012165.1	Forward	25792	26904	1113	371
B. burgdorferi	64b	lp38	vlsH2a	BBU64B_J0054	ACN24209.1	CP001419.1	Reverse	38518	38685	168	56
B. burgdorferi	64b	lp38	vlsH2b	BBU64B_J0055 (truncated seq.)	ACN24221.1	CP001419.1	Reverse	37583	38518	936	312
B. burgdorferi	64b	lp38	vlsH3c	--	--	CP001419.1	Forward	38252	38476	225	75
B. burgdorferi	B-17/2013	pGr-39_lp31	vlsH1	KSP41_05575	QXG44787.1	CP077736.1	Reverse	123	1247	1125	375
B. burgdorferi	B-17/2013	pGr-39_lp31	vlsH2 (fragment)	--	--	CP077736.1	Reverse	5003	5191	189	63
B. burgdorferi	B31	lp38	vlsH1a	--	--	AE000787.1	Reverse	38355	38522	168	56
B. burgdorferi	B31	lp38	vlsH1b	--	--	AE000787.1	Reverse	37930	38355	426	142
B. burgdorferi	B31	lp38	vlsH1c	--	--	AE000787.1	Reverse	37765	37926	162	54
B. burgdorferi	B31	lp38	vlsH1d	--	--	AE000787.1	Reverse	37421	37768	348	116
B. burgdorferi	B331	lp36	vlsH1	BHT49_06480	ATH10806.1	CP017216.1	Forward	28983	30089	1107	369
B. burgdorferi	B500	lp36	vlsH1	KGA77_04855	QYM87986.1	CP074057.1	Forward	28982	30088	1107	369
B. burgdorferi	Bb16-183	lp28-6	vlsH1	AB1P05_05860	XDA31913.1	CP161063.1	Reverse	121	1119	999	333
B. burgdorferi	Bb16-183	lp28-5	vlsH2a	AB1P05_06305 (mod.)	XDA31992.1 (mod.)	CP161066.1	Reverse	243	1235	993	331
B. burgdorferi	Bb16-183	lp28-5	vlsH2b	--	--	CP161066.1	Reverse	125	244	120	40
B. burgdorferi	JD1	lp28-6	vlsH1	BbJD1_Z01	WP_014540465.1	NC_017408.1	Reverse	122	1231	1110	370
B. burgdorferi	N40	lp38	vlsH1a	--	--	CP002237.1	Reverse	37558	37725	168	56
B. burgdorferi	N40	lp38	vlsH1b	--	--	CP002237.1	Reverse	36626	37558	933	311
B. burgdorferi	WI91-23	lp28-6	vlsH1	BBUWI9123_Z0001	WP_012666096.1	NC_012195.1	Reverse	90	1196	1107	369
B. burgdorferi	WI91-23	lp36	vlsH2	BBUWI9123_K0044	WP_012665966.1	NC_012189.1	Forward	28947	30050	1104	368
B. burgdorferi	WI91-23	lp38	vlsH3a	BBUWI9123_J0061	ACN55722.1	CP001458.1	Reverse	38351	38518	168	56
B. burgdorferi	WI91-23	lp38	vlsH3b	--	--	CP001458.1	Reverse	37419	38351	933	311

B. garinii	PBr	lp28-4	vlsH1a	BGAPBR_I0023	ACL34581.1	CP001304.1	Forward	10716	11816	1101	367
B. garinii	PBr	lp28-4	vlsH1b	--	--	CP001304.1	Forward	11820	11900	81	27
B. garinii	PBr	lp36	vlsH2	BGAPBR_K0044	ACL34505.1	CP001302.1	Reverse	31602	32795	1194	398
B. garinii	PBr	lp36	vlsH3	BGAPBR_K0022	ACL34581.1	CP001302.1	Forward	13807	14874	1068	356
B. valaisiana	VS116	lp28-3	vlsH1	BVAVS116_H0001	ACN52946.1	CP001440.1	Forward	191	1372	1182	394

[a] excluding stop codon
Yellow = common 5' frameshift
Orange = common 3' frameshift
Green = rearrangement within the vlsH region
